# Estimating Dengue Transmission Intensity from Sero-Prevalence Surveys in Multiple Countries

**DOI:** 10.1371/journal.pntd.0003719

**Published:** 2015-04-16

**Authors:** Natsuko Imai, Ilaria Dorigatti, Simon Cauchemez, Neil M. Ferguson

**Affiliations:** 1 MRC Centre for Outbreak Analysis and Modelling, Department of Infectious Disease Epidemiology, Imperial College London, London, United Kingdom; 2 Mathematical Modelling of Infectious Diseases Unit, Institut Pasteur, Paris, France; University of Oxford, UNITED KINGDOM

## Abstract

**Background:**

Estimates of dengue transmission intensity remain ambiguous. Since the majority of infections are asymptomatic, surveillance systems substantially underestimate true rates of infection. With advances in the development of novel control measures, obtaining robust estimates of average dengue transmission intensity is key for assessing both the burden of disease from dengue and the likely impact of interventions.

**Methodology/Principal Findings:**

The force of infection (*λ*) and corresponding basic reproduction numbers (*R_0_*) for dengue were estimated from non-serotype (IgG) and serotype-specific (PRNT) age-stratified seroprevalence surveys identified from the literature. The majority of *R_0_* estimates ranged from 1–4. Assuming that two heterologous infections result in complete immunity produced up to two-fold higher estimates of *R_0_* than when tertiary and quaternary infections were included. *λ* estimated from IgG data were comparable to the sum of serotype-specific forces of infection derived from PRNT data, particularly when inter-serotype interactions were allowed for.

**Conclusions/Significance:**

Our analysis highlights the highly heterogeneous nature of dengue transmission. How underlying assumptions about serotype interactions and immunity affect the relationship between the force of infection and *R_0_* will have implications for control planning. While PRNT data provides the maximum information, our study shows that even the much cheaper ELISA-based assays would provide comparable baseline estimates of overall transmission intensity which will be an important consideration in resource-constrained settings.

## Author Summary

With an estimated 390 million infections each year, dengue imposes a significant global public health burden. Yet estimates of the intensity of dengue transmission in different settings are still sparse, making it difficult to plan efficient control programs. Since many dengue infections have no symptoms, cases reported through hospitals are only a small proportion of true cases. The authors used seroprevalence surveys which can detect all past infections to estimate dengue transmission intensity in 22 countries. Estimates derived from data collected using cheaper diagnostic tests were comparable to those making use of more expensive tests, an important conclusion for surveillance in resource constrained countries. We found dengue transmission intensity varied up to 4-fold in endemic settings, with estimates showing some sensitivity to how many dengue infections were assumed to confer complete immunity.

## Introduction

Affecting more than one hundred countries with 2.5 billion people at risk and 50–100 million infections per year as estimated by the World Health Organisation (WHO), dengue is a global public health burden [1]. Estimates of global dengue distribution and transmission intensity (as quantified by either the force of infection—the per capita rate at which susceptible individuals acquire infection, or the basic reproduction number, (*R*_0_) remain ambiguous [2]. Infection with any of the four serotypes of dengue virus (DENV-1, 2, 3, and 4) can cause dengue fever with increased risk of more severe dengue with subsequent heterologous infections. Individuals develop protective monotypic immunity upon infection with a single serotype. Cross-reactive immunity is short-lived and the waning of antibodies below a threshold can facilitate antibody-dependent enhancement (ADE) upon secondary heterologous infection increasing the risk of more severe outcomes of dengue (such as dengue haemorrhagic fever (DHF) and shock syndrome (DSS)) [3–5]. The impact of cross-immunity and tertiary and quaternary infections are controversial. The estimated duration of short-term cross-protection varies widely from four months to 9 years [6], 5–12 months [7], 2 years [8], and 1–3 years [9]. However whether this protects against infection or clinically apparent disease is unknown. Therefore individuals may still contribute to onward transmission [8,10,11]. Clinically apparent tertiary and quaternary infections are rarely reported, and cannot be tested for retrospectively [10]. Wikramaratna *et al*. showed that tertiary and quaternary infections allows for the high seroprevalence at very young ages observed in Haiti [12] and Nicaragua [13] better than when assuming complete protection after two heterologous infections [10]. There are no antiviral therapies available as yet and disease control is restricted to vector control, community education and the development of an effective dengue vaccine.

Recent estimates of the global distribution of dengue and the resulting disease burden have refined our understanding, but remain controversial [2]. Shepard *et al*. highlight some of the difficulties in accurate dengue burden estimation including differences in surveillance systems leading to underestimation of dengue incidence, the lack of standardized reporting procedures or diagnostic criteria, and the lack of integration between private and public sectors [14]. Previous studies have attempted to estimate the burden of dengue and associated economic costs in South East Asia and South America by calculating expansion factors from systematic literature reviews, collation of existing data, and population-based cohorts [15–18]. In particular, Bhatt et al.’s estimate of 390 million dengue infections per year is three times higher than previous official WHO estimates, with India accounting for 34% of that total [2]. Motivated by previous work on malaria, the Bhatt et al. analysis relied on correlating their geographic niche-modelling based estimates of dengue presence with burden estimates derived from serological surveys. While an improvement on previous approaches, the fact that dengue infection induces serotype specific neutralising immunity weakens the parallels with malaria, in that the maximum number of dengue infections an individual can experience is strictly limited (while a person can experience dozens of malaria infections in their lifetime). Here we argue that obtaining robust estimates of the geographic variation in average dengue transmission intensity—as quantified by the basic reproduction number, *R*_0_ (the average number of secondary cases resulting from the introduction of a single infectious individual into a large susceptible population [19]), of each serotype—is key to improving the reliability of burden estimates. In addition, a quantitative understanding of variation in transmission intensity is essential to assessing the likely impact of interventions such as vaccine [20,21] or novel vector control measures [22–24].

However, with no standardised diagnostic method, challenging clinical diagnosis (Box 1) and highly variable surveillance systems, there is no consistent way to estimate global dengue transmission [25–27]. Dengue transmission is geographically highly heterogeneous, even down to very fine spatial scales [28]. Most model-based estimates of dengue transmission intensity and reproduction number have utilised case-notification data, which heavily depend on the quality of the surveillance system and the health infrastructure of the country in question [29–36]. Additionally, since the majority of dengue infections generate only mild symptoms, are asymptomatic, or are clinically diagnosed as a viral infection, even sensitive healthcare-based surveillance systems substantially underestimate true rates of infection [37,38]. Serological data are therefore invaluable in quantifying dengue transmission, in being able to identify both symptomatic and asymptomatic past infections and thus quantify infection prevalence and incidence in the population as a whole.

**Box 1. Main issues associated with current diagnostic methods.**

- Although highly accurate and sensitive, virus isolation and PCR can be time consuming and expensive and relies on sampling (and therefore detection) of symptomatic cases.
- Routinely used serological methods—IgM and IgG ELISAs—are unable to differentiate between the 4 dengue serotypes and are affected by cross-reactivity with other flaviviruses (e.g. yellow fever or Japanese encephalitis).
- IgG ELISAs are unable to differentiate between past, recent, and current infection [5].
- IgM ELISAs can be confounded by false positives and are only useful for a limited time post-infection [86].
- In secondary or later infections, serological diagnosis of the most recent infecting dengue serotype is difficult due to the presence of pre-existing cross-neutralising and cross-reactive antibodies [39,87].
- Serological protocols (e.g. thresholds used to define seropositivity) are not standardised across laboratories [26].
- Laboratory capacity and general public health infrastructure and surveillance systems vary widely within and between countries.

Here we utilise published age-stratified seroprevalence surveys and estimate the force of infection (*λ*) and corresponding basic reproduction number (*R*_0_) for dengue in a variety of settings. Due to the much lower costs, future seroprevalence studies are still likely to depend on IgM or IgG enzyme-linked immunosorbant assays (ELISAs) rather than the more labour intensive plaque-reduction neutralisation tests (PRNTs). The comparison of estimates derived from IgG, IE and PRNT data allows us to determine the usefulness of less expensive assays.

## Methods

### Literature search

**Fig 1:**
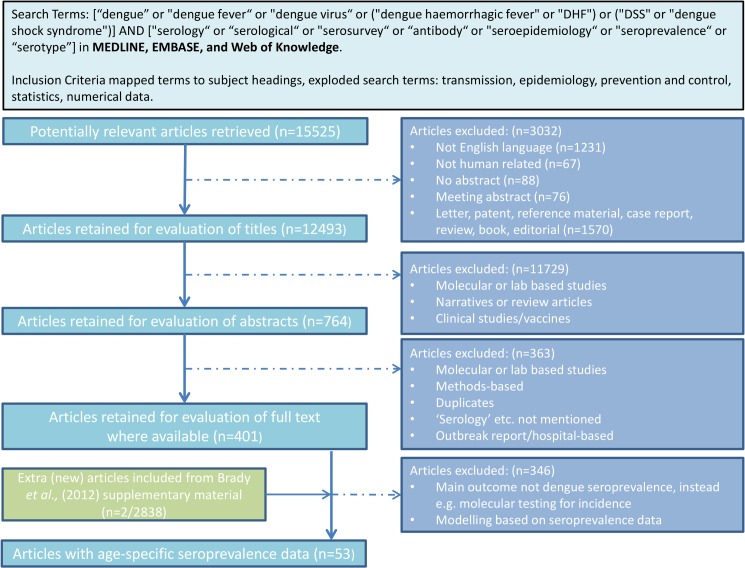
Flowchart describing the literature search process for dengue seroprevalence surveys.

We searched MEDLINE, EMBASE, and Web of Knowledge for publications reporting age-stratified dengue serological surveys. Fig 1 describes the search process and search terms used. Studies published before 1980 were not included in the analysis as we were interested in contemporary dengue transmission. Studies reporting age-specific seroprevalence for at least 5 age groups were included and categorised according to the assay type used. Studies reporting less than 5 age groups were excluded as these studies tended to have wide age groups where the mean seroprevalence did not accurately reflect the variability in seroprevalence within that age group. Data were extracted from published datasets where age-specific seroprevalence was tested by IgG ELISAs, inhibition ELISAs (IEs) or PRNTs. IgG and IE data are both non-serotype specific and we refer to them interchangeably.

### Estimating the force of infection (*λ*)

In the context of dengue, seroprevalence measures obtained with IgG ELISAs only give an indication of whether an individual has ‘ever’ been infected and do not differentiate between infecting serotypes or identify the number of past infections.

Since infection with one serotype only provides homologous immunity, a seropositive individual may still be susceptible to secondary heterotypic infection [39]. We fitted the single cross-sectional IgG datasets using a simple catalytic model (model A). The model assumes a constant infection hazard *λ*, with infection causing individuals of age *a* to move from a seronegative *x*(*a*) to a seropositive *z*(*a*) state [19].

Since some datasets appeared to have declining seroprevalence with age, we extended model A by assuming that protection could decay with age at a rate *α* (model B). Whenever yearly cross-sectional IgG data were available from the same location, these data were fitted using a time-varying catalytic model (model C) which allowed estimation of the periodicity (*T*), seasonal amplitude (*δ*) and within-year timing (*θ*) of dengue outbreaks, and the critical age (*A*_*crit*_) and scale (*S*) at which exposure levels change. See the Supporting Information for full details (S1 Text).

In order to fit serotype-specific PRNT data, we applied the multi-strain catalytic model developed by Ferguson *et al*. [40]. Different model variants were assessed, which explored different assumptions on serotype interactions. Model D1 assumed no serotype-interaction. Model D2 assumed that cross-protection or enhancement did not vary by serotype. Model D3 assumed that the magnitude of cross-protection or enhancement varied by the primary infection serotype. Last, model D4 assumed that the magnitude of cross-protection or enhancement depended on the serotype of the secondary infection.

Moreover, for comparison purposes, we fitted model A to PRNT data, having defined individuals with PRNT titres below the detection limit for all four dengue serotypes as seronegative and individuals with at least one PRNT titre over the detection limit as seropositive. Since assays differed between surveys, here the detection limit also varied from study to study.

We defined a beta-binomial likelihood for models A—C and a multinomial likelihood for models D1-D4. Models were fitted to the data using the Metropolis-Hasting Markov Chain Monte Carlo (M-H MCMC) algorithm using the R Statistical Package (version 3.1.0, R Development Core Team, Vienna, Austria) [41]. Full details are given in S1 Text.

### Estimating the basic reproduction number *R*_0*i*_

We assumed that dengue is at endemic equilibrium and that the force of infection *λ* is constant in time in all cases except model C. Unless otherwise stated, we assumed that all four serotypes of dengue were in circulation. Since IgG data contain no information on the infecting serotype, we assumed that the four dengue serotypes are equally transmissible and estimated a single reproduction number applicable to each serotype. For the PRNT data, since we were able to estimate serotype-specific forces of infection, we computed strain-specific reproduction numbers as described by Ferguson *et al*. [40].

We computed the reproduction numbers under two different assumptions on the number of infections required to obtain full protection against infection by any dengue serotype. This allows us to explore whether tertiary and quaternary infections contribute to transmission significantly. Under assumption 1 complete protection is obtained upon quaternary infection (all four infections contribute equally); whilst under assumption 2 complete protection is reached upon secondary infection (only primary and secondary infections are infectious). Under assumption 2 we were also able to incorporate cross-immunity leading to inhibition or enhancement of susceptibility to secondary infection. For each model variant other than B, we computed the serotype-specific basic reproduction number under assumptions 1 and 2. We only considered model B under assumption 1, as decay of immunity by definition allows an arbitrary number of infections to occur. Full details are given in S1 Text.

## Results

We identified 53 studies reporting age-specific seroprevalence from a total of 15,525 potentially relevant papers (Fig 1). Of these, 38 used non-serotype specific assays including IgG and inhibition ELISAs (IE). Only nine studies used PRNTs and five studies reported results from multiple assays. Excluding studies with less than 5 reported age groups from further analysis left a total of 30 surveys from 18 countries for IgG data, and 7 studies from 5 countries for PRNT data. 28 (out of 30) surveys from 17 countries were cross-sectional IgG seroprevalence surveys from a single year. The remaining 2 (out of 30) surveys were conducted in Nicaragua and combined provided 7 years’ worth of cross-sectional inhibition ELISA (IE) data. Most IgG surveys identified were conducted in 2000–2010 (23/30), while most PRNT surveys were conducted in the 1990s (4/7). Although recent serosurveys used commercial diagnostics, many studies used in-house assays. Tables 1 and 2 summarises the study and demographics of the datasets retained for analysis from the corresponding or closest year. All studies summarised in Table 1 were fitted using model A and B, and model C was also fitted to the two Nicaraguan datasets (Table 1). Models D1—D4 were fitted to studies summarised in Table 2.

**Table 1:** Summary of cross-sectional non-serotype specific datasets identified and associated demographics.

Country	Author	Survey Year	Region	Assay Type^+^	# Serotypes circulating	Age range sampled	N	Population size of study region (thousands)	Rural/Urban	% <15 years old	Models used
**Brazil**	Braga *et al*.[42]	2005/06	Recife	PanBio	4	5–65	2817	40	Urban	28	A and B
**Costa Rica**	Iturrino-Monge *et al*.[43]	2002/03	Puntarenas/San Jose	PanBio	4	1–10	206	358/1373	Urban	31.5	A and B
**Dominican Republic**	Yamashiro *et al*.[44]	2002	Santo Domingo	Focus Tech	4	0–60	1209	1887	Urban	35	A and B
**El Salvador**	Hayes *et al*.[45]	2000/01	Las Pampitas	CDC	NA	0–69	371	944	Rural	38	A and B
**FrenchPolynesia**	Deparis *et al*.[46]	1996	Teroma	In-house	4	0–21	169	16	Urban	34	A and B
**India**	Padbidri *et al*.[47]	1988/89	Andaman	HI/N	NA	0–40	2401	356	Rural	38	A and B
**Laos**	Vallée *et al*.[48]	2006	Vientiane	In-house	4	0–6	143	277	Urban	40	A and B
	Hiscox *et al*.[49]	2007/08	Khammouane	HI	4	0–90	1708	337	Rural		A and B
**Mayotte**	Sissoko *et al*.[50]	2006	Mayotte	Focus Tech	NA	2–55	1154	175	Whole island	41	A and B
**Mexico**	Brunkard *et al*.[51]	2004	Matamoros	PanBio	4	15–75	600	412	Urban	32	A and B
	Ramos *et al*.[52]	2005		Quantitative	4	5–65	131	412	Urban		A and B
**Pakistan**	Ali *et al*.[53]	Pre-2003^	Khyber Pakhtunkhawa	Cortez	NA	0–60	613	20000	Urban/rural	42	A and B
	Mahmood *et al*.[54]	2012	Lahore	NovaTech	NA	15–55	274	7566	Urban	35	A and B
**Papua New Guinea**	Senn *et al*.[55]	2007/08	Madang Province	PanBio	NA	0–25	577	493	Urban/rural	39	A and B
**Peru**	Hayes *et al*.[56]	1992	Loreto	In-house	2	0–60	1608	9	Urban/Rural/Jungle	38	A and B
	Reiskind *et al*.[57]	1996	Santa Clara	In-house	2	5–87	1225	2.4	Suburban	36	A and B
**Singapore**	Goh *et al*.[58]	1984	National	HI	4	0–40	425	2709	Urban	24	A and B
	Yew *et al*.[59]	2004	National	PanBio	4	18–74	4152	2709	Urban	19	A and B
	Yap *et al*.[60]	2007	National	PanBio	4	7–85	3939	2709	Urban	17	A and B
**Sri Lanka**	Malavige *et al*.[61]	Pre-2006^	Colombo district	PanBio	4	6–18	313	2309	Urban	25	A and B
	Tissera *et al*.[62]	2008	Columbo City	In-house	4	0–12	797	647	Urban	25	A and B
	Tam *et al*.[63]	2008	Colombo City	In-house	4	0–12	797	647	Urban	25	A and B
**Thailand**	Perret *et al*.[64]	2000	Bangkok	In-house	4	5–12	283	6355	Urban	24	A and B
	Tuntaprasart *et al*.[65]	2000	Ratchaburi	In-house	4	15–40	245	842	Urban	21	A and B
**USA**	Brunkard *et al*.[51]	2004	Brownsville	PanBio	NA	15–75	600	139	Urban		A and B
	Ramos *et al*.[52]	2005		Quantitative	NA	5–65	139	139	Urban	36	A and B
**Vietnam**	Bartley *et al*.[66]	1996/97	Dong Thap Province	PanBio	4	0–20	308	309	Urban/Rural	32	A and B
	Thai *et al*.[67]	Pre-2005^	Binh Thuan Province	MRL	4	7–14	961	1100	Rural	27	A and B
**Nicaragua** ^-^	Balmaseda *et al*.[68]	2001–03	Managua	IE	4	5–16	1971	2101	Urban	41	A and C
	Balmaseda *et al*.[13]	2004–07	Managua	IE	4	2–9	14182	2101	Urban	38	A and C

^ Survey date not given, noted as ‘pre-year of publication’. ^+^All assays were IgG or HI ELISAs. ^-^Cross-sectional surveys from multiple years (2001–2007).

**Table 2:** Summary of PRNT surveys identified and associated demographics.

Country	Author	Year	Region	Age Range (Yrs)	N	Serotypes^	Population size of study region (thousands)	Rural/Urban	% Aged <15 yrs	Models used
**Cuba**	Guzman *et al*.[69]	1983	Cerro	0–45	1295	2	125.5	Urban	26	A, D1—D4
	Guzman *et al*.[70]	1997/98	Santiago	0–95	1151	2	475.6	Urban	17.3	A, D1—D4
**Haiti**	Halstead *et al*.[12]	1996/99	Port au Prince	6–14	210	4	2000	Urban	43	A, D1—D4
**Indonesia**	Graham *et al*.[71]	1995	Yogyakarta	4–10	1837	4	421	Urban	34	A, D1—D4
**Peru**	Morrison *et al*.[72]	1999	Iquitos	5–60+	2524	2	350	Urban	34	A, D1—D4
**Thailand**	Sangkawibha *et al*.[73]	1980	Rayong	0–10	1009	4	53	Suburban	39.4	A, D1—D4
**Thailand**	Rodriguez-Barraquer *et al*.[74]	2010	Rayong	6–19	1647	4	230	Urban	19.3	A, D1—D4

*^Number of serotypes known to have been in circulation*.

**Fig 2:**
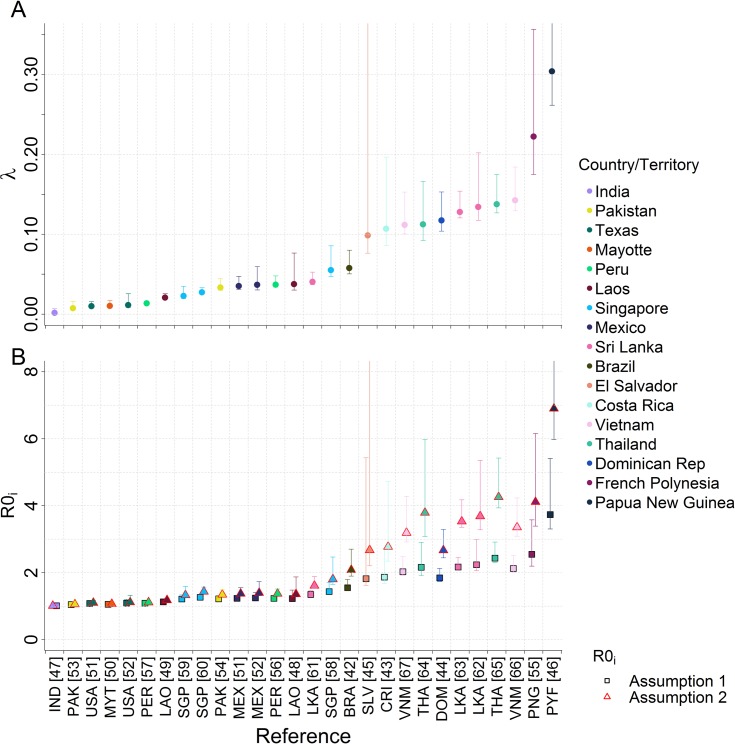
A) Force of infection and B) corresponding *R*0_*i*_ estimates of cross-sectional non-serotypes specific datasets fitted to Model A. Posterior median and 95% credible intervals shown.

Only an overall force of infection could be estimated from non-serotype specific IgG data. As expected, estimates of the force of infection varied widely between countries and, to a lesser extent, within countries (Fig 2A). Southeast Asian countries known to be hyper-endemic for dengue, such as Vietnam and Thailand, had a higher force of infection compared with most sites in the Americas [75]. Corresponding estimates of *R*_0*i*_ varied according to the assumptions made regarding host immunity (Fig 2B). Assuming that two heterologous infections are sufficient for complete immunity (Assumption 2) produced up to two-fold higher estimates of *R*_0*i*_ compared to when we assumed that quaternary infections are required for complete immunity (Assumption 1). However, *R*_0*i*_ estimates under these two assumptions converge as the estimated force of infection decreases.

**Fig 3:**
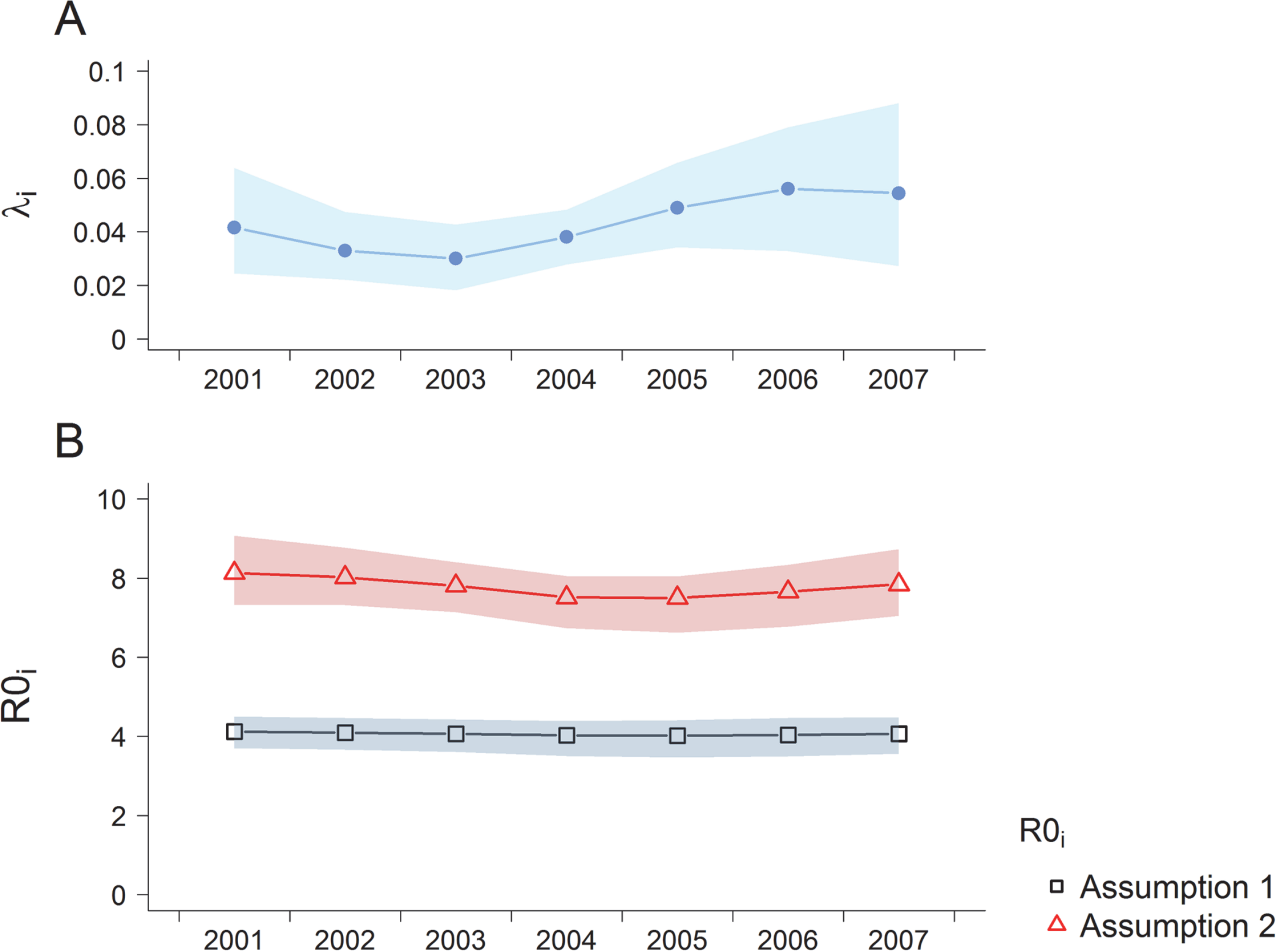
Estimated time-varying A) serotype-specific force of infection in individuals under the threshold age and B) *R*0_*i*_ derived by fitting Model C to Nicaraguan data (2001–2007). Posterior median and 95% credible intervals shown.

With age-structured serosurvey data from multiple sequential years (as was available for Nicaragua, Table S3), it is possible to estimate temporal and age-specific changes in exposure [13,68] (Fig 3A). We fitted a model (model C) to those data which allowed for the force of infection to vary sinusoidally over time and to change at (fitted) age threshold. We estimated that exposure increased in individuals over 3.9 years old (95% CI: 2.7–5.4 years), with the estimated force of infection during the study period (2001–2007) being 0.323 (95% CI: 0.261–0.377) above 3.9 years and 0.174 (95% CI: 0.118–0.280) below 3.9 years. These estimates represent the average total force of infection for all four serotypes in circulation. The force of infection was estimated to vary with a period of 8.8 years (95% CI: 1.3–12.5 years). Resulting estimates of *R*_0*i*_ (Fig 3B) showed the same dependence on immunity assumptions as the point estimates derived from single serosurveys (Fig 2), but interestingly showed less temporal variation than the force of infection estimates (Fig 3A).

**Fig 4:**
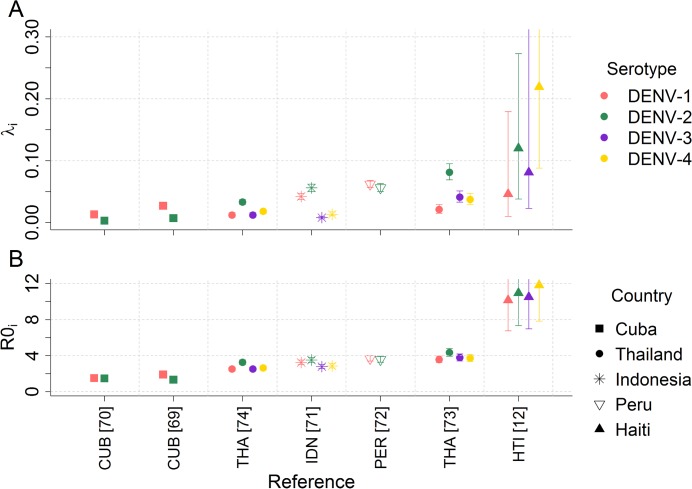
Serotype-specific estimates of A) force of infection, *λ*_*i*_, and B) *R*_0*i*_ estimates derived from PRNT datasets fitted to Model D2. Posterior median and 95% credible intervals shown.

PRNT data are serotype-specific, allowing us to estimate the force of infection (*λ*_*i*_) and basic reproduction number (*R*_0*i*_) for each serotype individually (Fig 4). Estimates varied widely between different surveys, again highlighting the heterogeneity of dengue transmission. Within the same survey, serotype-specific differences in transmission intensity were apparent, demonstrating how a certain serotype may be more dominant at any one time point. For example, for model D2, force of infection estimates for Haiti were 0.046 (95% CI: 0.010–0.179) for DENV-1 but 0.219 (95% CI: 0.088–0.445) for DENV-4.

Comparison of cross-protection or enhancement parameters under different assumptions allowed us to estimate the probable serotype causing primary and secondary infections. However, due to the wide credible interval of the estimated parameter, it is difficult to definitively determine the sequence of infections (Tables S5—S8 in S1 Text). For all datasets, the model fit improved when we assumed some level of inter-serotype interaction, demonstrating that inter-serotype interactions play an important role in dengue dynamics.

Interestingly, the serotype-specific estimates of the reproduction number did not scale linearly with the estimated values of the force of infection, although the relative order is maintained i.e. if *λ*_3_ < *λ*_4_ then *R*_03_ < *R*_04_. If one serotype dominates, as was the case in Haiti, changes in the force of infection of the other non-dominant serotypes marginally affect the estimates of the reproduction number of the non-dominating serotypes.

**Fig 5:**
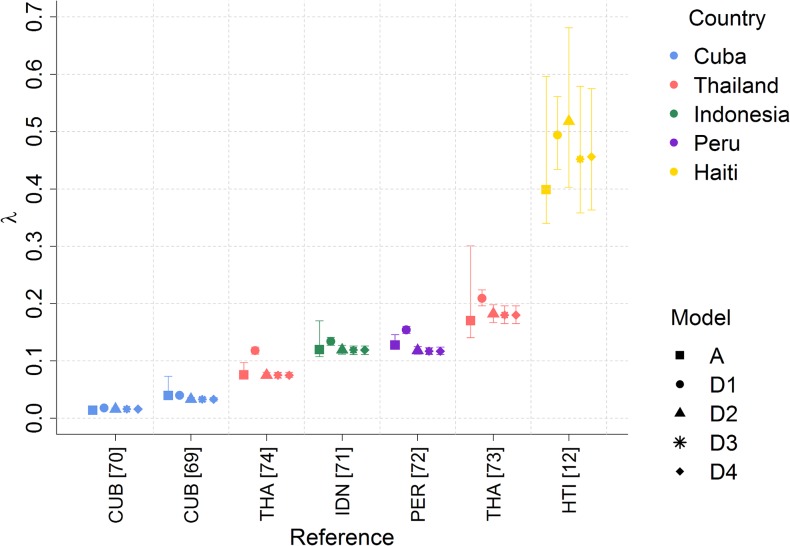
Total force of infection (λ) estimates (for all 4 serotypes) derived from PRNT datasets fitted to Models A (treating PRNT data as IgG data) and D1–D4. Models D2–D4 allow for cross-protection between serotypes. Posterior median and 95% credible intervals shown.

In order to compare the estimates of dengue force of infection derived from IgG and PRNT assays, we also analysed the PRNT data ignoring strain-specificity (*i*.*e*. treating PRNT data as if it were IgG data), by categorising individuals as ‘seronegative’ if their PRNT titers were negative for all serotypes, or seropositive if they tested positive for at least one serotype. We used the same thresholds for seronegativity used by each source study. The resulting force of infection estimates generated using model A were consistent with the sum of the individual serotype-specific λ estimates obtained from the full PRNT datasets. This consistency was highest when some level of inter-serotype interaction (cross-protection or enhancement) was allowed for (Fig 5).

## Discussion

From a literature review, we selected 39 studies reporting age-structured seroprevalence data obtained with IgG/IE (31 out of 39) or PRNT (8 out of 39) assays in 22 different locations from 1980 to 2010. From each dataset, we estimated dengue transmission intensity, quantified by the force of infection (λ) and the basic reproduction numbers (*R*_0*i*_). Overall, our estimates highlight the highly heterogeneous nature of dengue transmission in both space and time, and by serotype. Our analysis also highlights how the relationship between the force of infection and *R*_0*i*_ is affected by underlying assumptions about serotype interactions and immunity. The majority of our estimates of *R*_0*i*_ from 22 countries ranged from 1–4 (28 out of 28 and 24 out of 28 from model A fitted to IgG datasets under assumption 1 and 2 respectively, and 6 out of 7 from model D2 fitted to the PRNT surveys).

Dengue epidemiology differs between the Americas and Southeast Asia. Severe dengue predominantly affects children in Southeast Asia in contrast to the Americas where disease more often manifests in adults as the milder dengue fever [75]. However the changing demographics in Thailand (lower birth and death rates) have increased the average age of DHF suggesting that the epidemiology will continue to evolve [36]. However with the cross-sectional data we use in this study it is difficult to determine whether the higher force of infection in South East Asia is a reflection of the length of time dengue has been in circulation. The recent Phase III dengue vaccine trial conducted in several countries in Latin America showed that the forces of infection are highly heterogeneous across Latin America, with some countries comparable to South East Asia (Columbia and Honduras) and others having much lower forces of infection (Mexico and Puerto Rico) [76]. However, multiple cross-sectional surveys or cohort studies would be needed to estimate how forces of infection by age have changed over time. The low *R*_0*i*_ estimated in the Indian subcontinent is probably due to the lack of datasets from this region and the spatial heterogeneity of transmission within that large region. The one serosurvey from India used in our study was conducted in Andaman, an island with a low population density where we estimated a very low force of infection. It is likely that the epidemiology of dengue on Andaman is not representative of dengue epidemiology on the mainland.

Seroprevalence surveys have the benefit of not being affected by surveillance system sensitivity or case reporting rates, but still have several limitations (Box 1) [77,78]. A particular issue is the wide variation in the assays used between studies (Table 1). Optimally, one would assess the sensitivity of transmission intensity estimates to factors that varied between assays, such as the threshold used to define seronegativity. However, such an analysis requires access to the raw titer data which was not provided in any of the publications we reviewed. Additionally seroprevalence surveys sometimes use serum samples collected for a different purpose and therefore may not be representative of the population. Six out of the 37 studies used such samples: from blood banks [44], ante-natal clinics [64], hospitals [55,79,80], or residual samples from a different study [66]. Use of convenience samples can increase the volume of serological data produced, but the potential biases such sampling introduces must be taken into account when analysing such data.

Although we can only calculate a total force of infection across all serotypes from non-serotype specific data (such as surveys using IgG ELISA assays), such data are still sufficient for assessing heterogeneity in overall dengue transmission intensity between different populations. However as demonstrated by the variable serotype specific *λ*_*i*_ estimated from the PRNT data, even within the same population, the dominant serotype in circulation changes over time [8,81,82]. Furthermore, we found that estimates of *R*_0*i*_ varied between serotypes, suggesting serotypes (or genotypes) differ in their intrinsic transmissibility [40,74,82]. Therefore the assumption that all serotypes have identical *λ*_*i*_ required to estimate serotype-specific transmission intensity from IgG data must be regarded as a crude simplification. However, we found that non-serotype specific data does yield an estimate of the total force of infection from all serotypes consistent with the sum of serotype-specific forces of infection able to be derived from PRNT data, particularly when analysis of the latter allowed for inter-serotype interaction (cross-protection or enhancement) [8].

It is not possible to disentangle temporal from any age-dependent variation in exposure from single cross-sectional seroprevalence surveys, requiring broad assumptions to be made about such variation. Hence, for simplicity, we generally assumed constant transmission intensity over time when analysing single cross-sectional surveys. However, for Nicaragua [13,68], data from multiple sequentially conducted serosurveys were available, so we were able to estimate time and age-dependent changes in the force of infection. We found evidence of long term variation in transmission intensity over a timescale of 1–12 years, and that exposure levels changed with age, with children aged 4 or older having twice the exposure of those under that age. We suspect that this may be associated with school attendence, with children spending more time away from home leading to an increase in exposure if the majority of transmission is occuring outside the domestic environment [72]. This school-cohort effect has also been observed in Sri Lanka, conversely with a decrease in exposure, where Tam *et al*. estimated an age-varying force of infection of 0.154 (95% CI: 0.132–0.177) for 0.5–6 year olds and 0.087 (95% CI: 0.020–0.154) for children aged 6 years and above also demonstrating the existence of different transmission environments [63].

Our analysis has a number of additional limitations. First, in translating force of infection estimates into estimates of *R*_0*i*_ we rely on a model which assumes exposure is due to endemic transmission, meaning all resulting *R*_0*i*_ estimates are by definition greater than one. Clearly this is less appropriate for settings with low seroprevalence such as Texas (USA), where some or all of the seropositivity detected is due to imported cases rather than local transmission.

Second, estimates of transmission intensity (particularly *R*_0*i*_) are sensitive to assumptions about cross-protective immunity between serotypes—and most notably the extent to which tertiary and quaternary infections contribute to transmission. While there is increasing evidence that tertiary and quaternary infections occur [10,82], there is little quantitative data on the infectiousness of such infections relative to primary and secondary infections. Consistent with published theory [81], our estimates of *R*_0*i*_ were lower when we assumed tertiary and quaternary infections were as infectious as earlier infections (Assumption 1) than when we assumed complete immunity was acquired after secondary infection (Assumption 2). When one serotype had a very large force of infection relative to the other three serotypes (e.g. Haiti model 2: DENV-1 at 0.046 (95% CI: 0.010–0.179) compared to DENV-4 at 0.219 (95% CI: 0.088–0.445), then regardless of the value of *λ*_*i*_ of the remaining serotypes, all *R*_0*i*_ estimates were large and similar to each other. Thus it appears that the value of *R*_0*i*_ is dominated by very large *λ*_*i*_ and changes in the other three *λ*_*i*_ play a minimal role. This uncertainty has relevance for planning interventions [8,11,83], since *R*_0_ determines the coverage and effectiveness of vaccination or vector control measures required to achieve control of transmission [84]. The recent results from trials of the Sanofi live-attenuated chimeric vaccine [20,21] make this issue more pressing, since reliable estimates of transmission intensity—and of the health burden due to dengue—will be important in strategic planning and resource allocation for vaccination in different contexts.

Third, while PRNT assays are currently the gold standard for routine dengue serotyping, cross-reactivity means care must be taken when interpreting the results of serosurveys in areas where there is co-circulation of different flaviviruses or routine use of yellow fever or Japanese Encephalitis vaccine [3].

Finally, our literature search highlighted that use of serological surveys as a tool to assess transmission remains rare for dengue, with publications of outbreak reports and notified case incidence data being much more common. Generally, published models estimating dengue transmission risk have therefore used notification data, the reliability of which therefore heavily depend on the quality of the surveillance system [85]. Gaining a better global picture of the variation in transmission will improve both estimates of the disease burden caused by dengue and assist in control planning. We would therefore advocate much more widespread and routine use of serological surveys as a surveillance tool which provides invaluable data for an immunising infection such as dengue. While PRNT data provides the maximum information, our study shows that even the much cheaper ELISA-based assays would provide reasonable baseline estimates of overall transmission intensity.

## Supporting Information

S1 Text Supporting information file containing methods, results, and extra figures. (DOCX)
