# Supplementary material for: Estimating Dengue Transmission Intensity from Sero-Prevalence Surveys in Multiple Countries
Source: PLoS Negl Trop Dis. 2015 Apr 16;9(4):e0003719. doi: 10.1371/journal.pntd.0003719 (PMC4400108; doi:10.1371/journal.pntd.0003719)
Supplement: S1 Text — (DOCX) [file pntd.0003719.s001.docx]

**Title: Estimating dengue transmission intensity from sero-prevalence surveys in multiple countries – Supporting Information**

**Authors:** Natsuko Imai¹, Ilaria Dorigatti¹, Simon Cauchemez^2^, Neil M. Ferguson¹

**Affiliations: 1.** MRC Centre for Outbreak Analysis and Modelling, Department of Infectious Disease Epidemiology, Imperial College London, London, United Kingdom. **2.** Mathematical Modelling of Infectious Diseases Unit, Institut Pasteur, Paris, France.

**Contents:**

1. **Methods**
   1. Catalytic Models and Associated data types
   2. Calculating the strain-specific basic reproduction number
   3. Justification of assay type in context of IgG cross-reactivity with Japanese encephalitis
2. **Results**
   1. Decay of protection model (model B)
   2. Figures

Fig. S3: Comparison of $R_{0i}$by continent. Constant λ model (model A) fit to IgG and PRNT data.

Fig. S4: Model fits from the constant λ model (model A) fit to IgG data

Fig. S5: Model fits from the decay of protection model (model B) fit to IgG data

Fig. S6: Model fits from the time-varying λ model (model C) fit to Nicaraguan data (2001-2007)

Fig. S7: Model fits from the constant λ model (model A) fit to PRNT data

Fig. S8-11: Model fits from the multi-serotype models (models D1 – D4) fit to PRNT data

- 1. Tables

Table S1: Summary results where the constant λ model (model A) was fitted to the cross-sectional non-serotype specific datasets.

Table S2: Summary results where the antibody decay model (model B) was fitted to the cross-sectional non-serotype specific datasets.

Table S3: Summary results where the time-varying model with seasonality (model C) was fitted to yearly cross-sectional non-serotype specific data from Nicaragua.

Table S4: Summary results where the constant λ model (model A) was fitted to PRNT data.

Table S5 – S8: Summary results where the multi-strain model (models D1 – D4) were fitted to PRNT data.

Table S9: DIC comparison of different model variants (A, D1 – D4) for serotype-specific PRNT datasets.

# 1. Methods

## 1.1 Catalytic Models

**Data Type: Single cross-sectional IgG ELISA surveys**

In the context of dengue infection we assume that upon infection individuals in age group $i$ move from being seronegative to seropositive. We denote the force of infection (also called the infection hazard) by $\lambda$; the proportions of the population of age *a* which are seronegative and seropositive as $x(a_{i})$and $z(a_{i})$, respectively (Figure S1). Since IgG data are not serotype-specific and infection may occur with any of the (up to) four serotypes, we assume that an IgG seropositive individual is susceptible to secondary heterotypic infections.


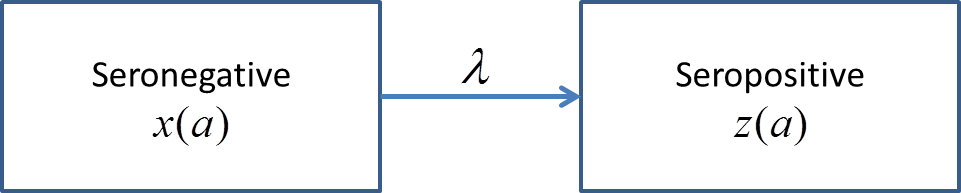


**Figure S1: Compartmental model showing the flow of individuals in a catalytic model.**

***Model A: Constant force of infection***

Assuming a constant force of infection due to all 4 serotypes, *λ*, the proportion seropositive (IgG positive) in age group $i$ at age *a*, $z(a_{i})$ is given by:

$z(a_{i})=1-\exp\left[-\lambda a\right]$ [1]

where $\lambda$ is the force of infection and *a* is the age in years.

***Model B: Constant force of infection and decay of protection***

We now additionally assume that protection decays at a rate *α* (Figure S2), moving people back to the seronegative class.

**
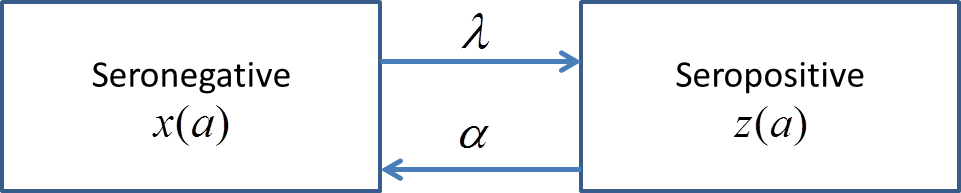
**

**Figure S2: Compartmental model showing the flow of individuals in a catalytic model where individuals may lose seropositivity/antibodies.**

Then the proportion of IgG seropositive individuals in age group $i$ at age *a* (in years) is given by:

$$\begin{matrix} \frac{dz}{da}=\lambda(1-z)-\alpha z \\ =\lambda-(\lambda+\alpha)z. \end{matrix}$$

Assuming $\lambda$ and $\alpha$ are constant, integrating gives:

[2]

**Data Type: Yearly cross-sectional IgG ELISA surveys**

***Model C: Time-varying force of infection with seasonality***

We extend model A to allow the force of infection to vary over time. We assume that the force of infection$\lambda$ has a constant component $\lambda_{0}$and a time-varying component given by a sinusoidal function with periodicity *T*, amplitude *δ* and phase *θ* :

We then also allow for exposure to change with age, by introducing a critical age $A_{crit}$ at which exposure levels change. Below that age, exposure is reduced by a scaling factor *S* relative to individuals over that age. For individuals younger than the critical age $(a<A_{crit})$the seroprevalence at age $a$ and time $t$ is given by:

For individuals aged equal to or above the critical age $(a\geq A_{crit})$, seroprevalence is given by:

[3]

We estimate parameters$\lambda_{0}$, $\delta$, $\theta$, $T$, $S$, and $A_{crit}$.

Models A-C are fitted to the IgG data from each available dataset using a Metropolis-Hastings Markov Chain Monte-Carlo algorithm with a beta-binomial likelihood. We assume that the probability of testing seropositive in each age group is beta-binomially distributed:

$$X_{i}\sim BetaBinomial\left(N_{i},p_{i},\gamma\right)$$

where $N_{i}$ is the total number of individuals in age group $i$, $p_{i}$ is the probability of testing seropositive (or the proportion in that age group seropositive), and $\gamma$ represents over-dispersion.

The likelihood is given by:

$L\left(p_{i}\right)=\left(\begin{matrix} N_{i} \\ X_{i} \end{matrix}\right)\frac{B\left(N_{i}+a,N_{i}-X_{i}+b\right)}{B\left(a,b\right)}$, where B is the beta function with standard arguments *a* and *b*.

We re-parameterise the beta distribution in terms of its mean ($m$) and variance ($v$):

$m=a/\left(a+b\right)$ and $v=ab/\left[\left(a+b\right)^{2}\left(a+b+1\right)\right]$ respectively.

We then define the over-dispersion parameter $\gamma$ as:

$\gamma=v/\left[m\left(1-m\right)\right]$.

Then the beta arguments become:

$a=m\left(1/\gamma-1\right)$ and $b=\left(1-m\right)\left[1/\gamma-1\right]$.

Here we assign $m$ = $p_{i}$ from equations [1]-[3] for models A-C respectively, and estimate *γ*.

Substituting the above into the likelihood this becomes:

$L\left(p_{i}\right)=\left(\begin{matrix} N_{i} \\ X_{i} \end{matrix}\right)\frac{B\left(X_{i}+m\left(1/\gamma-1\right),N_{i}-X_{i}+\left(1-m\right)\left[1/\gamma-1\right]\right)}{B\left(m\left(1/\gamma-1\right),\left(1-m\right)\left[1/\gamma-1\right]\right)}$.

Ignoring the constant, the log-likelihood for one age group is:

$LnL\left(p_{i}\right)=\log\left\{B\left(X_{i}+m_{i}\left(1/\gamma-1\right),N_{i}-X_{i}+\left(1-m_{i}\right)\left[1/\gamma-1\right]\right)\right\}-\log\left\{B\left(m_{i}\left(1/\gamma-1\right),\left(1-m_{i}\right)\left[1/\gamma-1\right]\right)\right\}$ .

So total log-likelihood across all age groups is:

$LnL\left(p\right)=\sum_{i} \log\left\{B\left(X_{i}+m_{i}\left(1/\gamma-1\right),N_{i}-X_{i}+\left(1-m_{i}\right)\left[1/\gamma-1\right]\right)\right\}-\log\left\{B\left(m_{i}\left(1/\gamma-1\right),\left(1-m_{i}\right)\left[1/\gamma-1\right]\right)\right\}$where $X_{i}$ is the number of individuals testing positive among those tested in age group *i*, $N_{i}$is the total number of individuals tested in age group *i*, $\gamma$ represents the degree of over-dispersion, and $m$ is the predicted proportion of seropositive individuals in that age group.

The predicted proportion of seropositive individuals in each age group is calculated by taking the average seroprevalence within each age group. For example for age group 5-9 years, seroprevalence at each age 5, 6, 7 etc. would be computed and the mean value taken as the seroprevalence for that age group. Ideally one would integrate over the entire age group, however this was computationally expensive.

**Data Type: Cross-sectional PRNT surveys**

Since dengue exists as four distinct serotypes, individuals may be seropositive for one serotype but seronegative for the other three serotypes. For example an individual may have a primary infection with DENV-1 (DENV-1 seropositive), then upon secondary infection with e.g. DENV-2, that individual becomes DENV-2 seropositive, still remaining DENV-1 seropositive. We then estimated the serotype-specific force of infection following Ferguson *et al.* [1] under the following assumptions:

***Model D1: No interaction between circulating serotypes***

Here we assume complete serotype independence. We assumed absence of antibody-dependent enhancement (ADE), no cross-protection, no change in susceptibility and no change in transmissibility following primary infection.

Under these assumptions the proportion seronegative against all dengue serotypes,$(x(a,t))$ is given by:

The proportion seropositive against strain $i$only is given by:

$\begin{matrix} w_{i}(a,t)=\left[\exp\left[-\int_{0}^{a} \sum_{k\neq i} \lambda_{k}(a-\tau,t-\tau)d\tau\right]\right]\left[1-\exp\left[-\int_{0}^{a} \lambda_{i}(a-\tau,t-\tau)d\tau\right]\right] \\ =x(a,t)\left[\exp\left[\int_{0}^{a} \lambda_{i}\left[1+\delta\sin\left(2\pi\left\{\frac{t-\tau}{T}+\theta\right\}\right)\right]d\tau\right]-1\right] \\ =x(a,t)\left[\exp\left[\left\{\lambda_{i}a-\frac{\lambda_{i}\delta T}{2\pi}\left(-\cos\left[2\pi\left(\frac{t-a}{T}+\theta\right)\right]+\cos\left[2\pi\left(\frac{t}{T}+\theta\right)\right]\right)\right\}\right]-1\right] \end{matrix}$ [4]

where $\lambda_{i}$ is the force of infection of strain $i$*,*$T$ is the periodicity in years, $\delta$ is the seasonal amplitude, $\theta$ is the phase shift, $t$ is the chronological time in years and $a$ is the age in years.

***Models D2-D4: Assuming interaction between serotypes***

The following models assume interaction between serotypes mediated by cross-immunity. We define$\rho_{ij}$to be susceptibility of an individual to infection with serotype *j* following infection with serotype *i* , relative to the susceptibility of an individual who has never been infected with dengue.

Ferguson et al [1] showed that the proportion of the population at age $a$ and time $t$*,* seropositive for strain $i$ and seronegative for each other serotype $j$ in circulation, $(j\neq i)$*,* $w_{i}(a,t)$, is given by:

$w_{i}(a,t)=x(a,t)\int_{0}^{a} \lambda_{i}(a-\tau,t-\tau)\exp\left[-\int_{0}^{\tau} \sum_{k} (\rho_{ik}-1)\lambda_{k}(a-\tau',t-\tau')d\tau'\right]d\tau$ [5]

where we assume that the force of infection is given by :

[6]

Substituting the definition of the force of infection given in equation (6) into equation (5) we obtain:

where $\lambda_{i}$ is the force of infection of strain $i$*,*$T$ is the periodicity in years, $\delta$ is the seasonal amplitude, $\theta$ is the phase, $t$ is the chronological time in years and $a$ is the age in years.

Finally, evaluating the integral between 0 and *a* gives: [7]

Here $x\left(a,t\right)$ is the proportion seronegative (completely unexposed to any strain of dengue), which is explicitly given by:

**Model D2:** We assume that susceptibility enhancement-inhibition $\rho_{ij}$ is identical for all strain combinations. We estimate 5 parameters: a force of infection for each serotype $\lambda_{k}$ $(k=1,...,4)$and one susceptibility parameter $\rho=\rho_{ij}$ for all $i,j=1,...,4$.

**Model D3:** We assume that susceptibility enhancement-inhibition is dependent only on the primary infecting strain$(\rho_{i\cdot})$. We estimate 8 parameters: a force of infection for each serotype $\lambda_{k}$ and a susceptibility enhancement-inhibition term for each primary infecting serotype $\rho_{k\cdot}$.

**Model D4:** We assume that susceptibility enhancement-inhibition is dependent only on the secondary infecting strain$(\rho_{\cdot j})$. We estimate 8 parameters: a force of infection for each serotype $\lambda_{k}$and a susceptibility enhancement-inhibition term for each secondary infecting serotype $\rho_{\cdot k}$.

Given a seroprevalence survey of $N$individuals at time $t_{0}$*,* the $N_{k}$ individuals in each age class $k$can be classified into: $n_{xk}$ the number unexposed (seronegative against any strain, PRNT < cut off defined in the study), $n_{ik}$ the number monotypically exposed against serotype $i$ (PRNT for serotype $i$*>* cut off defined and PRNT < cut off defined in the study for the remaining serotypes)*,* and $(N_{k}-\text{ }n_{xk}-\text{ }\sum_{i}n_{ik})$ multi-typically exposed (PRNT > cut off defined in the study for 2 or more serotypes)*.* The multinomial log-likelihood is then given by:

$L(\lambda_{ik})=\sum_{k=1}^{m} \left[n_{xk}\ln[x(a_{k},t_{0})]+\sum_{i} n_{ik}\ln[z_{i}(a_{k},t_{0})]+\left(N_{k}+n_{xk}+\sum_{i} n_{ik}\right)\ln\left[1-x(a_{k},t_{0})-\sum_{i} z_{i}(a_{k},t_{0})\right]\right]$ [8]

where the proportion seropositive and seronegative in each age group was calculated by taking the average seroprevalence within each age group. For example for age group 5-9 years, seroprevalence at each age 5, 6, 7 etc. would be computed and the mean value taken as the seroprevalence for that age group. Ideally one would integrate over the entire age group, however this was computationally expensive.

Models D1 – D4 were fitted to PRNT data using the Metropolis-Hastings Markov Chain Monte Carlo algorithm using the multinomial log-likelihood defined in equation (8). Since the available PRNT data are all cross-sectional seroprevalence surveys from a single year, we assume no seasonality and set$\delta=0$.

## 1.2 Estimating the basic reproduction number, *R*_0_

For each model, we compute the strain-specific basic reproduction number $R_{0i}$under two different assumptions:

1. Tertiary and quaternary infections possible – here we can only analytically derive an expression for $R_{0i}$ in the case that there are no cross-immunity mediated interactions between serotypes.
2. Individuals develop complete immunity to all dengue serotypes after secondary infection – in this case we can explore different assumptions about cross-immunity.

**Assumption 1:** Tertiary and quaternary infection possible.

When tertiary and quaternary infections are possible, we can only estimate $R_{0i}$ assuming there are no cross-immunity mediated interactions between serotypes. Thus estimates cannot be derived for models D2-D4.

Following Ferguson et al. [1], the serotype–specific basic reproduction number under assumption 1 is given by:

$R_{0i}=\frac{(d\lambda_{i}(t)/dt)/(\sigma\lambda_{i}(t))+1}{1-\int_{0}^{\infty} f(a')z_{i}(a',t)da'}$ [9]

where *σ* is the reciprocal of the infectious period (1/6 days) [2,3],$f(a)$ is the probability density function of the age distribution of the population and $z_{i}(a)$ is the proportion seropositive to serotype *i* at age *a*.

$\lambda(t)$ is given by:

$\lambda(t)=\lambda_{0}\left[1+ \delta\text{sin}\left(2\pi\left\{\frac{t}{T}+ \theta\right\}\right)\right]$ [10]

for Model C where we allow the force of infection to vary with time.

Assuming temporal changes in the force of infection are relatively small ($d\lambda/dt<<\sigma\lambda$), for models A, B, and D1 equation (9) reduces to:

Assuming that the serotypes are equally transmissible (and thus that the force of infection for each serotype is a quarter of the overall force of infection for dengue when four serotypes are in circulation, *λ*), the serotype-specific proportion of seropositive individuals of age a, $z_{i}(a)$, is given by equations (11)–(15) for models A-D1 respectively:

Model A: [11]

Model B: [12]

Model C:

For $a<A_{crit}$:

[13]

For $a\geq A_{crit}$:

[14]

Model D1:

[15]

**Assumption 2: Complete immunity after secondary infection**

If only primary and secondary infections can occur, we can relax the assumption of no cross-immunity between serotypes. Following Ferguson et al.[1], the serotype–specific basic reproduction number for models A – C is then given by

And for Models D1 – D4 is given by

Here $x(a,t)$ is the proportion seronegative at age *a* and time *t* and  is the relative infectiousness of someone infected with serotype $j$ following infection with serotype $i$(relative to a primary infection), and other terms are as defined previously. We set = 1 since this parameter cannot be estimated from serological data alone. $\lambda(t)$ is given in equation (10) for model C where we allow the force of infection to vary with time. Assuming temporal changes in the force of infection are relatively small ($d\lambda/dt<<\sigma\lambda$), for models A-B this reduces to

And for Models D1 – D4:

For Models A, C and D1 we set $\rho_{\text{ji}}=1$, while for models D2 – D4 we estimate the interaction parameters $\rho_{ji}$. Expressions for$z_{j}(a)$ are given below:

Model A:

Model C:

For $a<A_{crit}$

For $a\geq A_{critical}$:

For models D1 and D2-D4 the proportion of population seropositive for strain $j$only are given in equation (7).

We do not consider models combining both decay of protection and serotype interactions, as derivation of closed-form expressions for $R_{0i}$ proved intractable in this case.

For all calculations, $f(a)$ or the probability density function of the age distribution of the population was calculated from demography data corresponding to each study year – either from the UN population estimates, or where available the national census data of the corresponding study region.

## 1.3 Justification of assay type in context of IgG cross-reactivity with Japanese encephalitis

Of the studies that we included in our work, there were 6 countries where Japanese encephalitis cases occur (Laos, Sri Lanka, India, Thailand, Vietnam, and Singapore). Each author has justified their use of assay in their study context which is summarised below.

Singapore:

Yew *et al.,* Goh *et al*, Yap *et al* [4–6]*.-* Although the PanBio ELISA can be cross-reactive with JE, the incidence of JE in Singapore is very low in comparison to dengue which is endemic. They therefore conclude that the impact of false positives on the observed dengue seroprevalence would be minimal.

Sri Lanka:

- Tam *et al*. [7] specifically tested how the seropositivity against JE would affect dengue estimates using JE vaccination history as a proxy. They found no evidence for JE having an effect and conclude that the majority of past infections detected by the ELISA were dengue.

- Malavige *et al.* [8] cite the high specificity and sensitivity of the PanBio assay making it unlikely that JE was affecting the results, but go on to specify that false positives cannot be completely ruled out.

- Tissera *et al*. [9] surveyed in a known dengue area and so state that the impact of JE would be minimal.

India:

Padbidri *et al.* [10] tested samples in conjunction with neutralizing antibodies and so cross-reactivity with JE can be ruled out.

Thailand:

- Perret *et al*. [11] tested all samples with IgM and IgG ELISAs for JE as well and excluded cross-reactivity in dengue positive samples by calculating the ratio between JE and dengue IgG antibody.

- Tuntaprasart *et al*. [12] conducted a post-outbreak survey and therefore contribution of JE is likely to be minimal.

Laos:

- Vallee *et al*. [13] differentiate between recent JE and dengue infection. However they are unable to differentiate past infections. However they state that dengue infections appeared to be more frequent than JE.

- Hiscox *et al*. [14] categorized their results as: if a sample was positive for both JE and dengue this was considered flavivirus positive. If the sample produced a 2-fold higher titre to the homologous virus they were categorized as DENV positive or JEV positive only.

# 2. Results

## 2.1 Model B: Decay of protection model

The joint antibody decay rate (or decay of protection) was low when estimated across all non-serotype specific datasets at 0.020 (95% CI: 0.014 – 0.030). Allowing for decay of protection slightly increased the estimated force of infection for each dataset (Table S2). Of the non-serotype specific datasets examined, all 17 countries had more than one serotype in circulation in the past. As antibodies can be life-long, the variability in antibody levels between older age groups will inevitably be small. This may mask or change actual age-related changes in the data making accurate serological testing over a wide age range crucial. Ideally we would estimate decay of serotype-specific protection since serotype-specific antibody titres are important in terms of cross-protection and enhancement. However IgG data cannot differentiate between infecting serotypes so the antibody decay rates estimated here must be treated cautiously.

**Figure S3: Comparison of basic reproduction numbers by continent A) Assumption 1 tertiary and quaternary infections possible, and B) complete immunity after secondary infection. Model A fit to IgG data and PRNT data, having re-categorised them as ‘seropositive’ (PRNT > cut-off for at least one serotype) and ‘seronegative’ (PRNT < cut-off for all serotypes).**

**
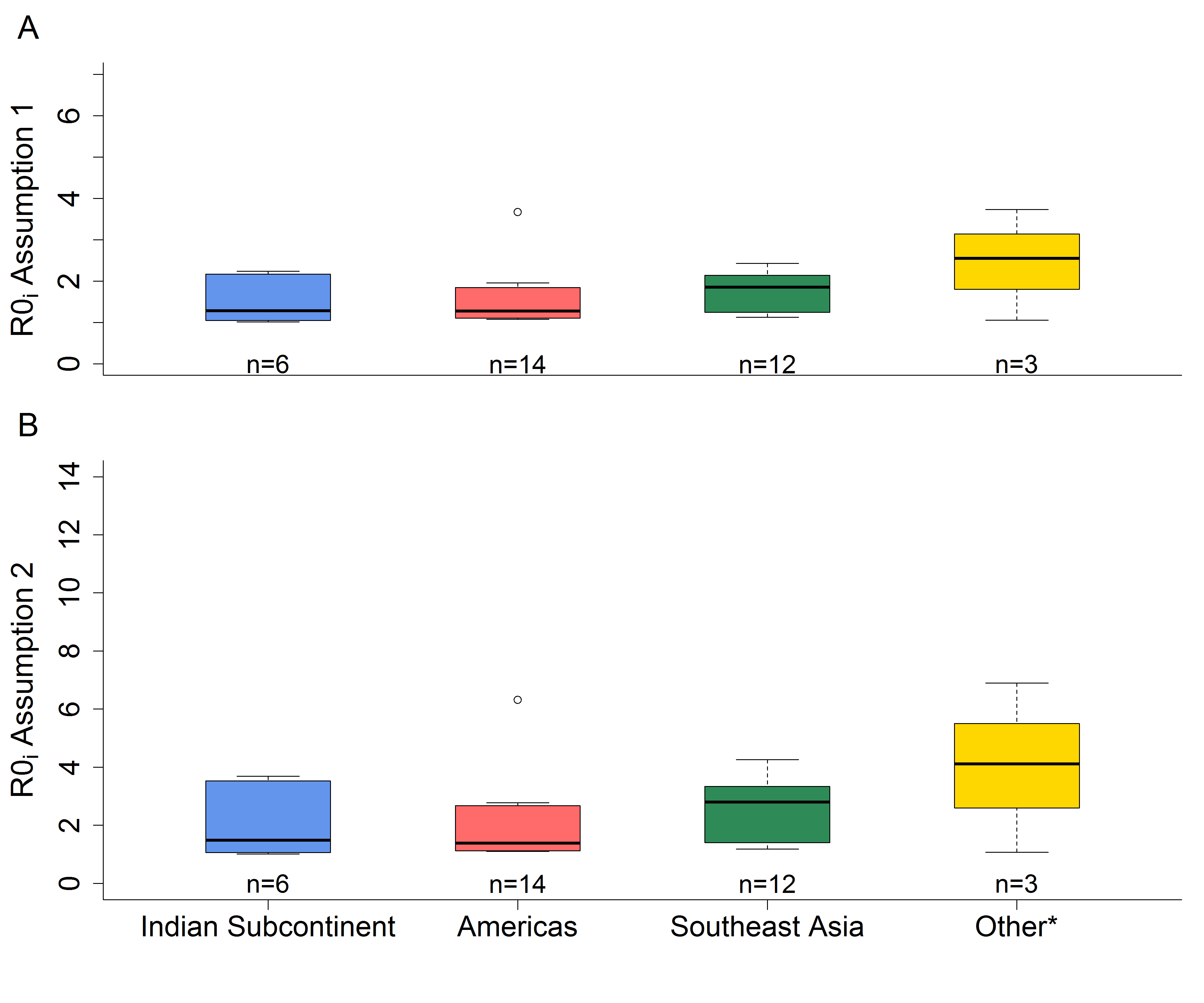
***Other: Oceania (Papua New Guinea and French Polynesia) and Africa (Mayotte).

**Figure S4: Model fits from the constant λ model (model A) fit to IgG data (points). 95% exact confidence intervals around data points, posterior median (line) and 95% credible intervals (shaded area) shown.**

**
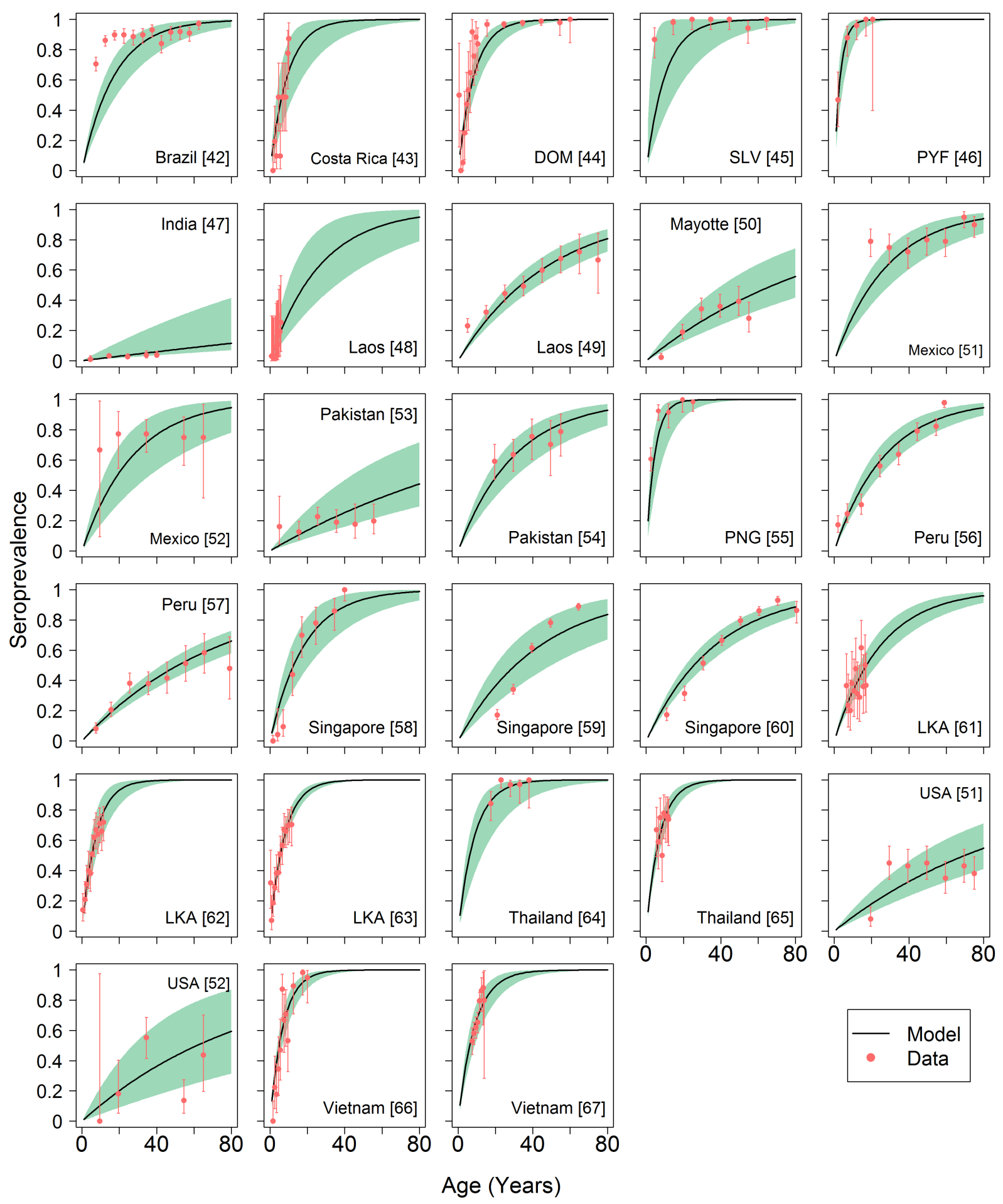
**

* DOM = Dominican Republic, SLV = El Salvador, PYF = French Polynesia, PNG = Papua New Guinea, LKA = Sri Lanka. [Ref] refers to reference list in main text.

**Figure S5: Model fits from the decay of protection model (model B) fit to IgG data (points). Posterior median (line) and 95% credible interval (shaded area) shown.**

**
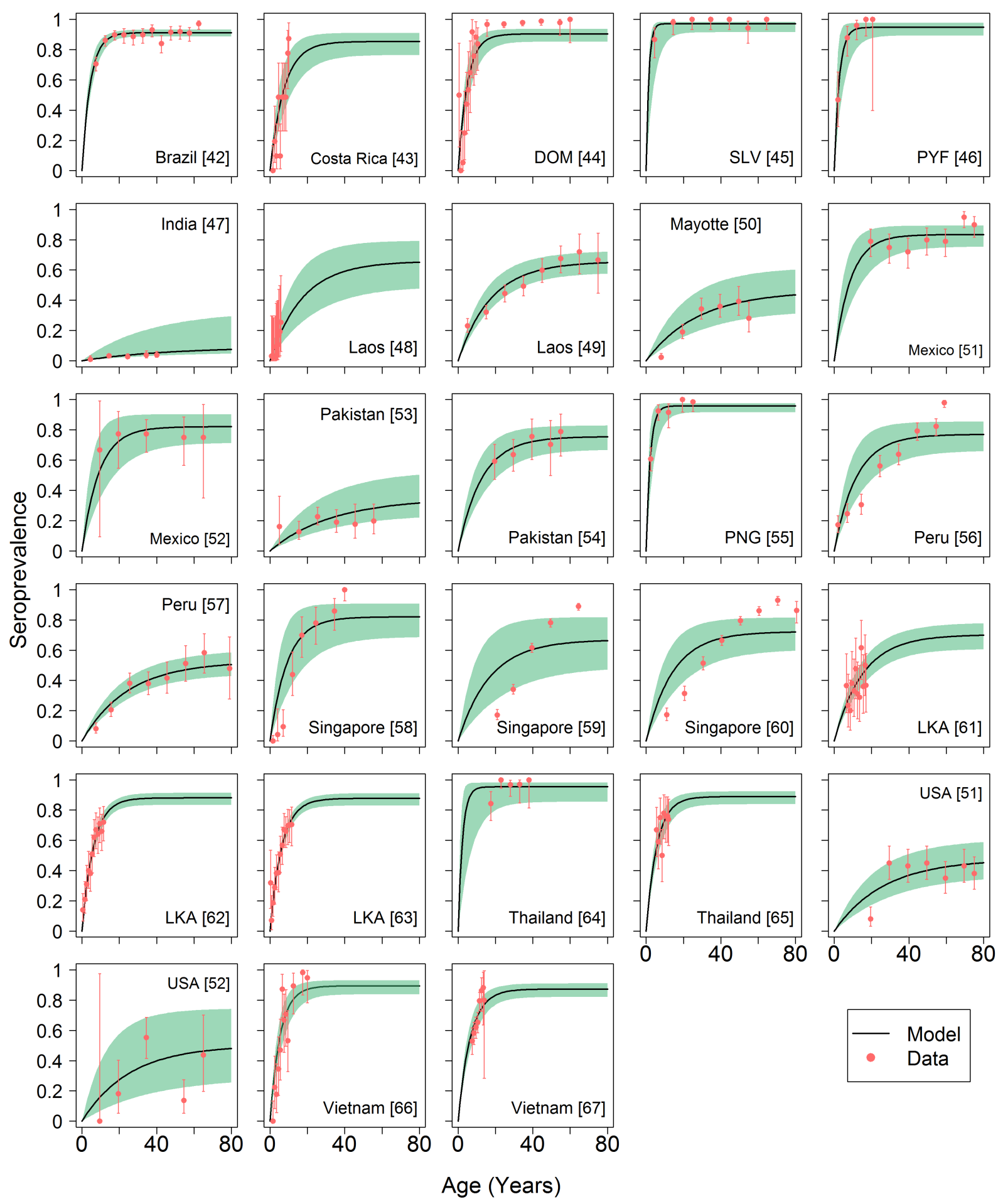
**

* DOM = Dominican Republic, SLV = El Salvador, PYF = French Polynesia, PNG = Papua New Guinea, LKA = Sri Lanka. [Ref] refers to reference list in main text.

**Figure S6: Observed (dots) and estimated (line) cross-sectional seroprevalence in Nicaragua from 2001 to 2007 using the age-threshold model with seasonal force of infection (model C).** **95% exact confidence intervals around data points, p** **posterior median (line) and 95% credible intervals (dashed lines) shown.**


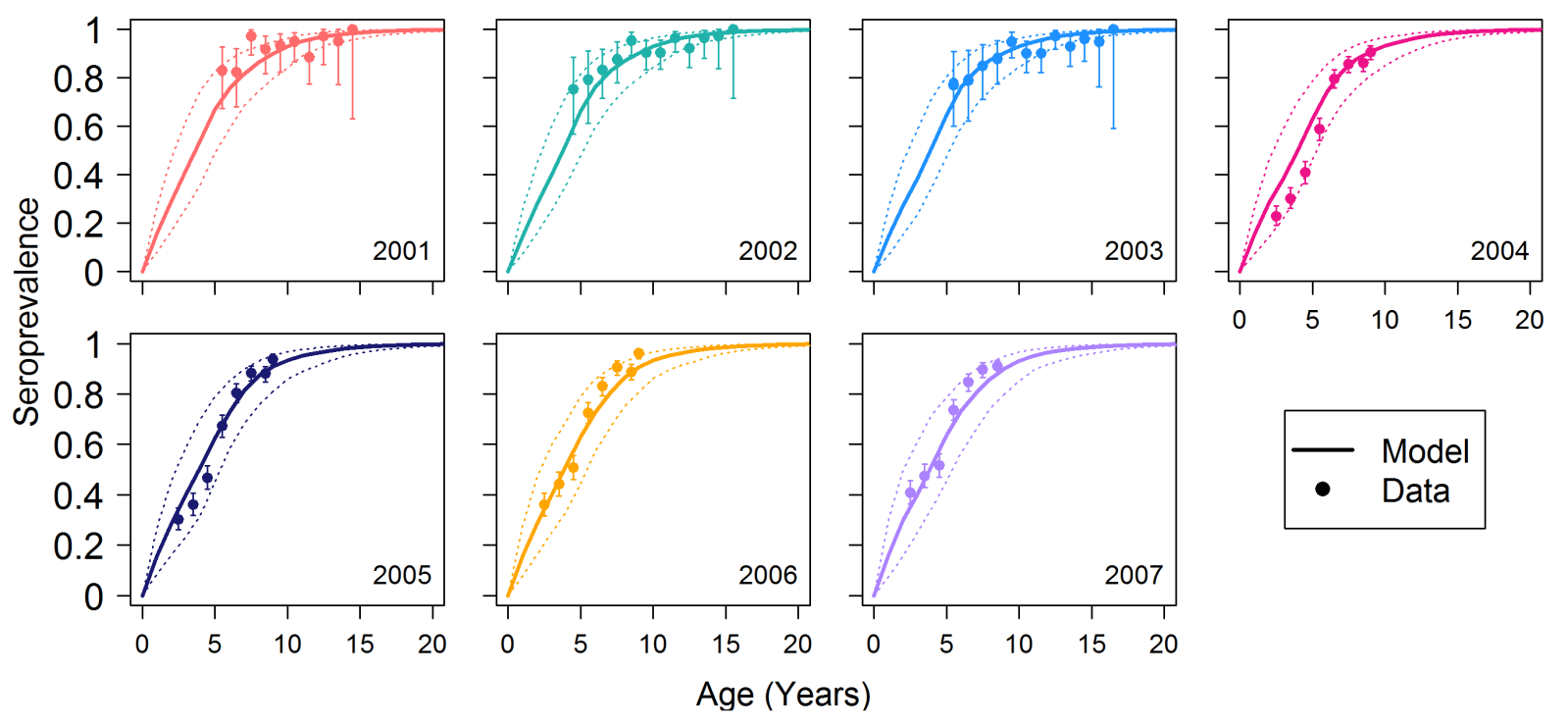


**Figure S7: Model A fit to PRNT data (points), having re-categorised them as ‘seropositive’ (PRNT > cut-off for at least one serotype) and ‘seronegative’ (PRNT < cut-off for all serotypes). 95% exact confidence intervals around data points, posterior median (line) and 95% credible intervals (shaded area) shown.**

**
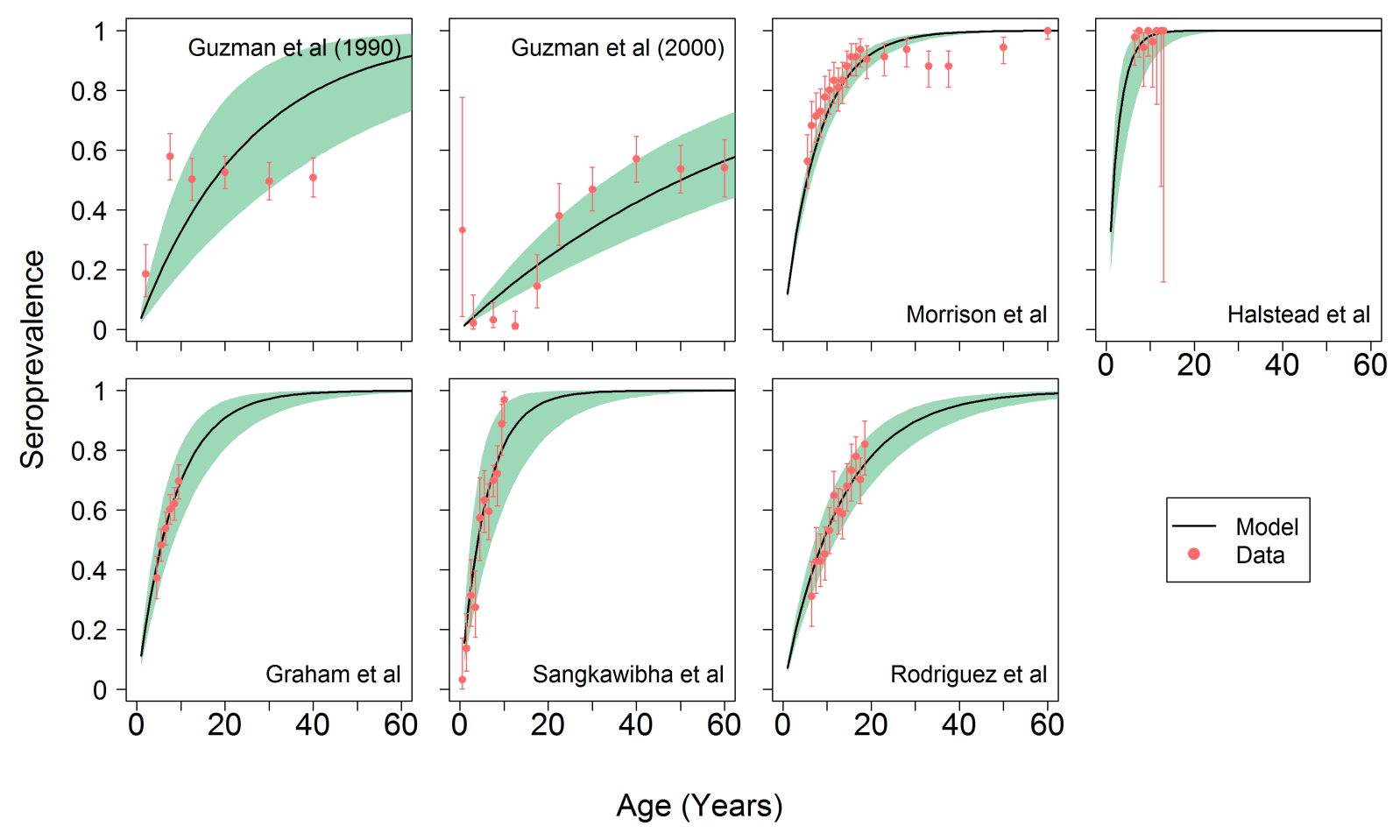
**

**Figure S8: Model fits from the multi-serotype model (D1) fit to PRNT data (points). 95% exact confidence intervals around data points, posterior median (line) and 95% credible interval (shaded area) shown.**


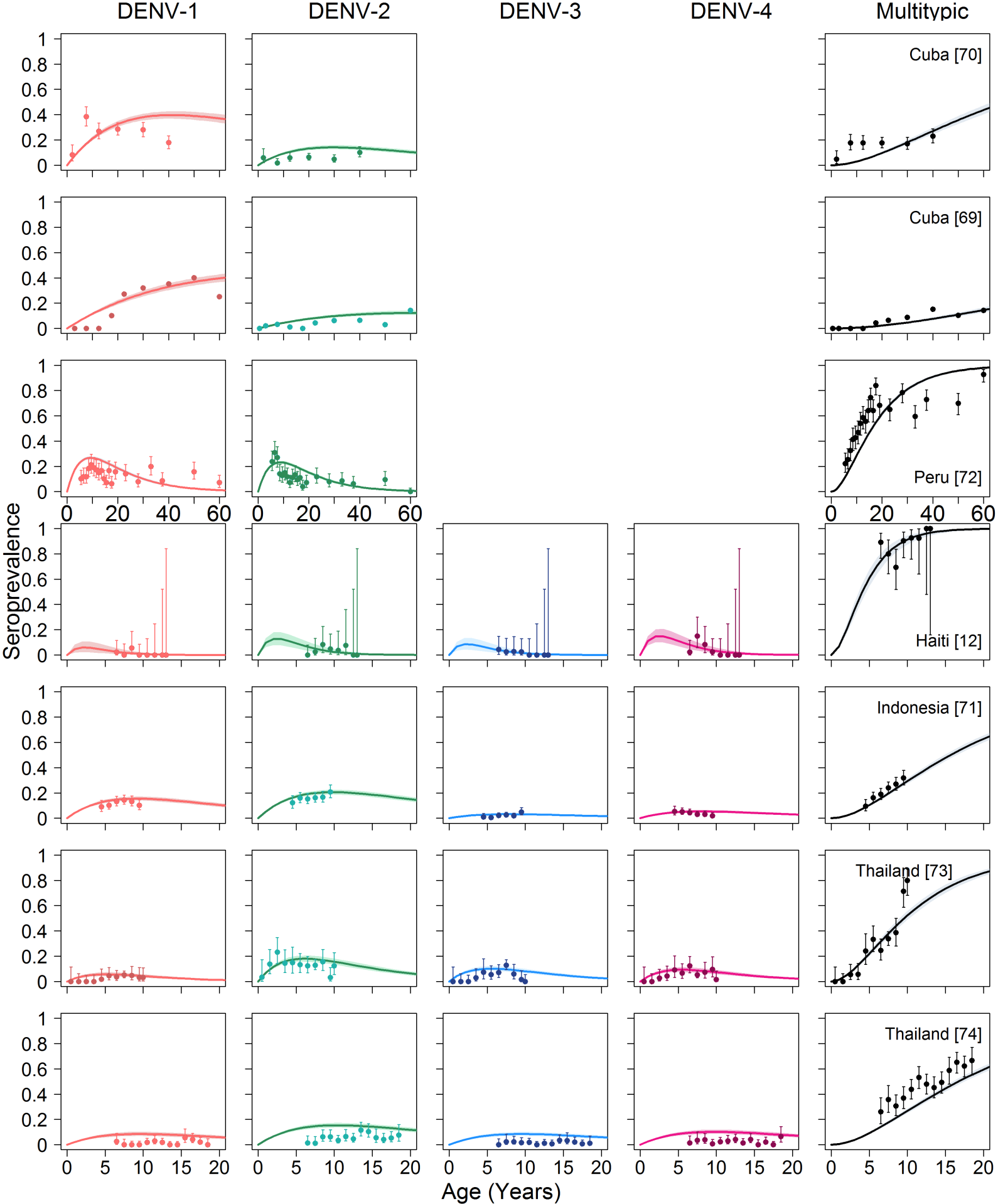
*Multitypic (right-most column) defined as multi-typically infected with more than one serotype. PRNT > cut-off point for ≥2 serotypes. [Ref] refers to reference list in main text.

**Figure S9: Model fits from the multi-serotype model (D2) fit to PRNT data (points). 95% exact confidence intervals around data points, posterior median (line) and 95% credible interval (shaded area) shown.**

**
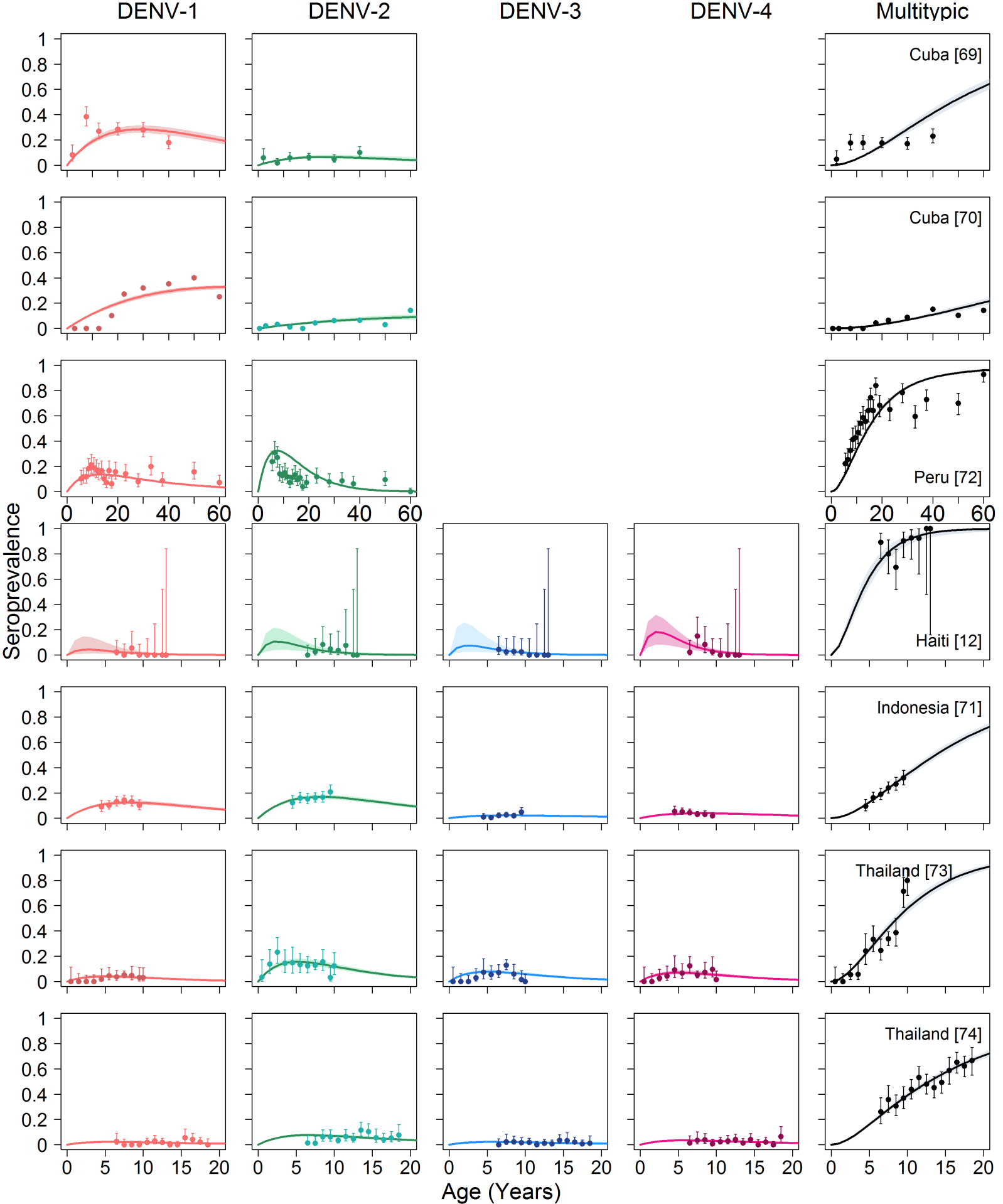
**

*Multitypic (right-most column) defined as multi-typically infected with more than one serotype. PRNT > cut-off point for ≥2 serotypes. [Ref] refers to reference list in main text.

**Figure S10: Model fits from the multi-serotype model (D3) fit to PRNT data (points). 95% exact confidence intervals around data points, posterior median (line) and 95% credible interval (shaded area) shown.**

**
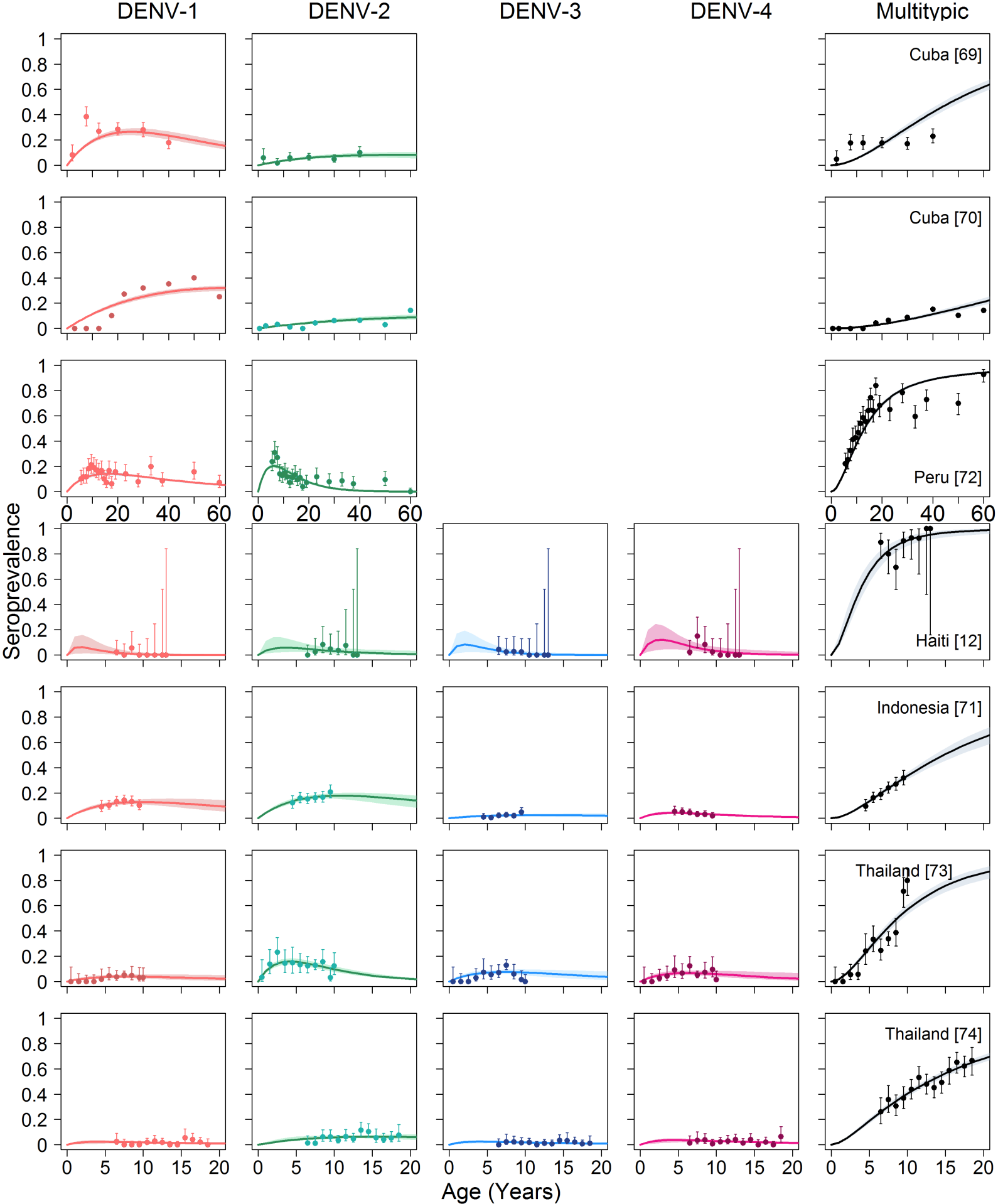
**

*Multitypic (right-most column) defined as multi-typically infected with more than one serotype. PRNT > cut-off point for ≥2 serotypes. [Ref] refers to reference list in main text.

**Figure S11: Model fits from the multi-serotype model (D4) fit to PRNT data (points). 95% exact confidence intervals around data points, posterior median (line) and 95% credible interval (shaded area) shown.**

**
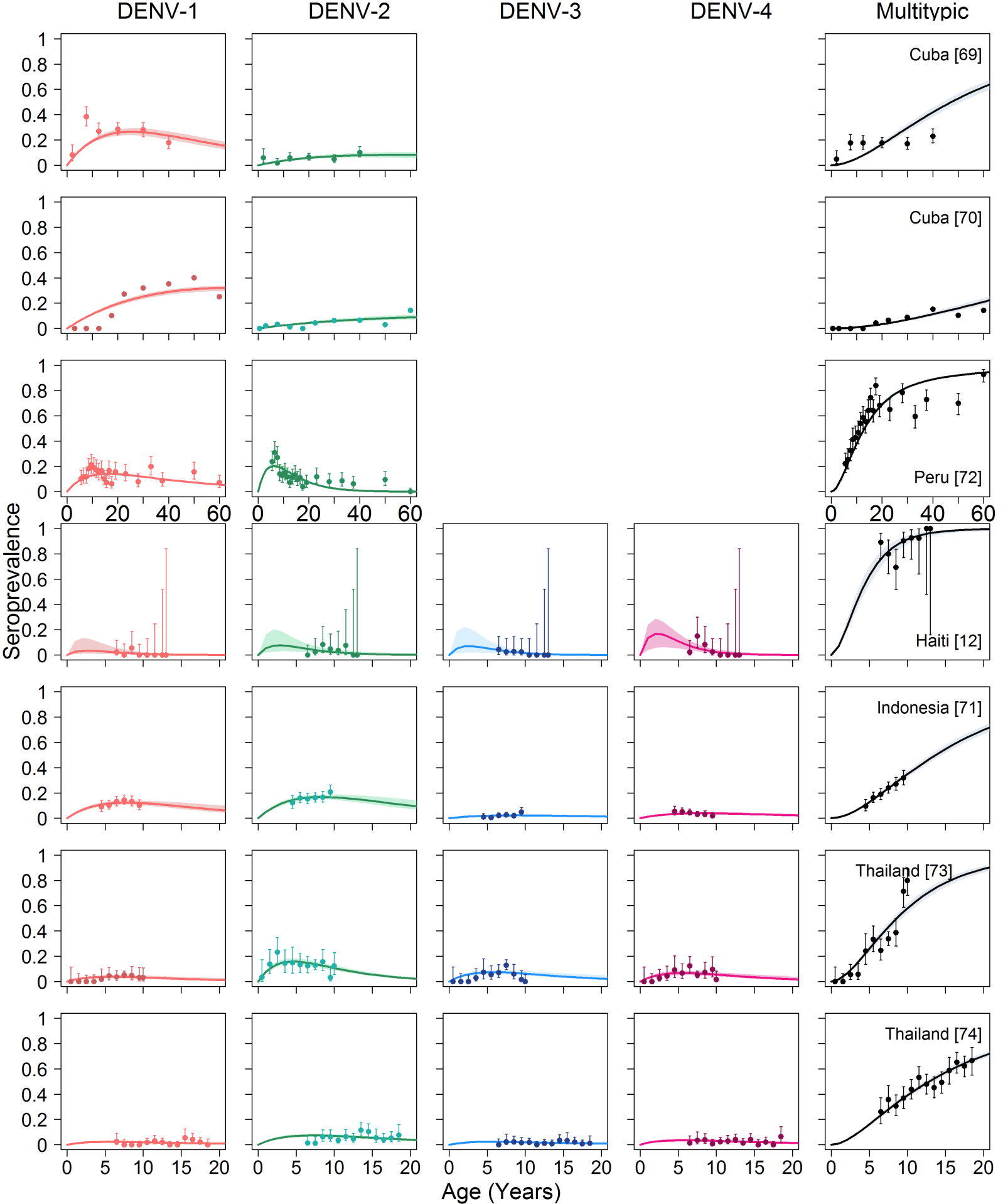
**

*Multitypic (right-most column) defined as multi-typically infected with more than one serotype. PRNT > cut-off point for ≥2 serotypes. [Ref] refers to reference list in main text.

**Table S1: Summary estimates from the fit of cross-sectional non-serotype specific datasets using model A.**

|  |  | Force of Infection $\lambda$ | Over-dispersion $\gamma$ | $R_{0i}$(95% CI) | |  |  |
| --- | --- | --- | --- | --- | --- | --- | --- |
| Country | **Author** | **(95% CI)** | **(95% CI)** | **Assumption 1** | **Assumption 2** | **LnL** | **DIC** |
| Brazil | Braga *et al.*[15] | 0.058 (0.050-0.080) | 0.155 (0.116-0.346) | 1.55 (1.47-1.80) | 2.08 (1.90-2.70) | -1056.2 | 2114.9 |
| Costa Rica | Iturrino-Monge *et al.*[16] | 0.107 (0.086-0.197) | 0.329 (0.264-0.555) | 1.87 (1.68-2.73) | 2.77 (2.35-4.72) | -115.8 | 234.2 |
| Dominican Republic | Yamashiro *et al.*[17] | 0.117 (0.104-0.153) | 0.087 (0.050-0.264) | 1.84 (1.74-2.13) | 2.67 (2.44-3.29) | -229.9 | 462.2 |
| El Salvador | Hayes *et al. (2)*[18] | 0.099 (0.076-0.430) | 0.443 (0.299-0.799) | 1.82 (1.62-5.43) | 2.67 (2.21-10.66) | -47.6 | 98.1 |
| French Polynesia | Deparis *et al.*[19] | 0.304 (0.261-0.463) | 0.044 (0.016-0.396) | 3.73 (3.30-5.41) | 6.90 (5.97-10.42) | -51.6 | 105.4 |
| India | Padbidri *et al.*[10] | 0.002 (0.001-0.007) | 0.010 (0.004-0.239) | 1.01 (1.01-1.04) | 1.01 (1.01-1.05) | -336.4 | 676.9 |
| Laos | Vallee *et al.*[13] | 0.037 (0.030-0.076) | 0.043 (0.018-0.231) | 1.23 (1.18-1.48) | 1.36 (1.28-1.87) | -46.4 | 95.4 |
|  | Hiscox *et al.*[20] | 0.021 (0.019-0.026) | 0.033 (0.022-0.126) | 1.13 (1.12-1.16) | 1.18 (1.16-1.23) | -1093.7 | 2190.3 |
| Mayotte | Sissoko *et al.*[21] | 0.010 (0.009-0.017) | 0.056 (0.033-0.241) | 1.06 (1.05-1.09) | 1.07 (1.06-1.12) | -544.6 | 1092.0 |
| Mexico | Brunkard *et al.*[22] | 0.035 (0.031-0.047) | 0.093 (0.061-0.300) | 1.23 (1.21-1.32) | 1.37 (1.32-1.55) | -286.3 | 575.1 |
|  | Ramos *et al.*[23] | 0.037 (0.030-0.059) | 0.133 (0.073-0.439) | 1.24 (1.20-1.41) | 1.40 (1.31-1.74) | -78.2 | 158.7 |
| Pakistan | Ali *et al.*[24] | 0.007 (0.006-0.016) | 0.063 (0.035-0.308) | 1.05 (1.04-1.10) | 1.05 (1.05-1.14) | -302.0 | 606.9 |
|  | Mahmood *et al.*[25] | 0.033 (0.030-0.044) | 0.040 (0.018-0.253) | 1.22 (1.19-1.29) | 1.34 (1.30-1.49) | -174.7 | 352.0 |
| Papua New Guinea | Senn *et al.*[26] | 0.222 (0.175-0.357) | 0.116 (0.053-0.521) | 2.55 (2.19-3.58) | 4.11 (3.39-6.15) | -175.7 | 353.4 |
| Peru^ | Hayes (1) *et al.*[27] | 0.037 (0.034-0.048) | 0.061 (0.042-0.189) | 1.37 (1.34-1.51) | 1.37 (1.34-1.51) | -828.1 | 1658.9 |
|  | Reiskind *et al.*[28] | 0.013 (0.013-0.016) | 0.014 (0.007-0.083) | 1.11 (1.11-1.14) | 1.11 (1.11-1.14) | -675.7 | 1354.2 |
| Singapore | Goh *et al.*[6] | 0.055 (0.047-0.086) | 0.229 (0.166-0.507) | 1.43 (1.37-1.71) | 1.80 (1.65-2.47) | -143.4 | 289.3 |
|  | Yew *et al.*[4] | 0.023 (0.020-0.035) | 0.147 (0.099-0.422) | 1.21 (1.18-1.34) | 1.33 (1.27-1.59) | -2273.7 | 4549.9 |
|  | Yap *et al.*[5] | 0.027 (0.025-0.033) | 0.041 (0.027-0.146) | 1.26 (1.25-1.33) | 1.44 (1.40-1.57) | -2084.4 | 4171.7 |
| Sri Lanka | Malavige *et al.*[8] | 0.040 (0.037-0.053) | 0.034 (0.018-0.139) | 1.35 (1.31-1.47) | 1.61 (1.54-1.88) | -206.8 | 416.4 |
|  | Tissera *et al.*[9] | 0.134 (0.118-0.202) | 0.160 (0.124-0.320) | 2.24 (2.06-2.99) | 3.69 (3.28-5.35) | -509.7 | 1022.1 |
|  | Tam *et al.*[7] | 0.128 (0.120-0.154) | 0.026 (0.015-0.089) | 2.17 (2.09-2.45) | 3.53 (3.35-4.18) | -504.8 | 1012.4 |
| Thailand | Perret *et al.*[11] | 0.112 (0.092-0.166) | 0.120 (0.050-0.607) | 2.16 (1.91-2.91) | 3.79 (3.08-5.98) | -46.8 | 95.6 |
|  | Tuntaprasart *et al.*[12] | 0.137 (0.127-0.175) | 0.038 (0.019-0.175) | 2.43 (2.30-2.92) | 4.26 (3.93-5.42) | -175.0 | 352.8 |
| USA (Texas) | Brunkard *et al.*[22] | 0.010 (0.009-0.016) | 0.086 (0.056-0.279) | 1.08 (1.07-1.12) | 1.10 (1.09-1.17) | -381.9 | 766.7 |
|  | Ramos *et al.*[23] | 0.011 (0.009-0.026) | 0.226 (0.149-0.578) | 1.09 (1.07-1.21) | 1.12 (1.08-1.32) | -84.1 | 170.6 |
| Vietnam | Bartley *et al.*[29] | 0.142 (0.129-0.184) | 0.088 (0.056-0.243) | 2.12 (2.02-2.50) | 3.36 (3.09-4.23) | -147.0 | 296.6 |
|  | Thai *et al.*[30] | 0.112 (0.101-0.153) | 0.093 (0.063-0.285) | 2.02 (1.91-2.45) | 3.19 (2.92-4.27) | -606.3 | 1215.3 |
| Nicaragua* | Balmaseda *et al.*[31,32] | 0.218 (0.214 – 0.223) | 0.016 (0.009 – 0.028) | 3.22 (3.16 – 3.27) | 6.42 (6.27 – 6.58) | -8184.2 | NA |

^Only 2 serotypes in circulation, calculation adjusted accordingly, i.e. assuming complete immunity upon secondary infection (assumption 2). *Model A fit to Nicaraguan data for comparison. Assumption 1: tertiary and quaternary infections possible, assumption 2: complete protection after secondary infection.

**Table S2: Summary results where all non-serotype specific datasets were fitted together using the antibody decay model (model B) to estimate an overall shared antibody decay rate.**

| Country | Author | $\alpha$(95% CI) | $\lambda$ (95% CI) | $\gamma$ (95% CI) | $R_{0i}$Assumption 1 | LnL | Combined LnL | DIC |
| --- | --- | --- | --- | --- | --- | --- | --- | --- |
| Brazil | Braga *et al.*[15] | 0.020  (0.014 – 0.030) | 0.203 (0.161 – 0.267) | 0.010 (0.001-0.039) | 2.32 (2.07-2.60) | -1063.0 | -13086.8 | 26254.3 |
| Costa Rica | Iturrino-Monge *et al.*[16] |  | 0.115 (0.072 – 0.178) | 0.158 (0.046-0.375) | 1.59 (1.36-1.95) | -116.3 |  |  |
| Dominican Republic | Yamashiro *et al.*[17] |  | 0.186 (0.128 – 0.258) | 0.164 (0.067-0.326) | 1.89 (1.60-2.26) | -306.3 |  |  |
| El Salvador | Hayes *et al. (1)*[18] |  | 0.702 (0.217 – 0.986) | 0.150 (0.015-0.596) | 5.11 (2.20-7.21) | -332.2 |  |  |
| French Polynesia | Deparis *et al.*[19] |  | 0.356 (0.172 – 0.811) | 0.131 (0.010-0.529) | 2.93 (1.89-5.47) | -41.6 |  |  |
| India | Padbidri *et al.*[10] |  | 0.002 (0.001 – 0.009) | 0.007 (0.000-0.251) | 1.01 (1.01-1.04) | -42.2 |  |  |
| Laos | Vallee *et al.*[13] |  | 0.038 (0.021 – 0.068) | 0.019 (0.001-0.134) | 1.16 (1.09-1.30) | -204.6 |  |  |
|  | Hiscox *et al.*[20] |  | 0.037 (0.028 – 0.053) | 0.025 (0.006-0.108) | 1.16 (1.13-1.22) | -492.7 |  |  |
| Mayotte | Sissoko *et al.*[21] |  | 0.017 (0.010 – 0.031) | 0.071 (0.019-0.288) | 1.07 (1.04-1.12) | -478.3 |  |  |
| Mexico | Brunkard *et al.*[22] |  | 0.098 (0.058 – 0.179) | 0.058 (0.013-0.219) | 1.47 (1.28-1.82) | -537.9 |  |  |
|  | Ramos *et al.*[23] |  | 0.090 (0.049 – 0.187) | 0.044 (0.001-0.288) | 1.43 (1.23-1.88) | -388.8 |  |  |
| Pakistan | Ali *et al.*[24] |  | 0.011 (0.007 – 0.021) | 0.036 (0.003-0.245) | 1.05 (1.03-1.10) | -89.6 |  |  |
|  | Mahmood *et al.*[25] |  | 0.060 (0.041 – 0.097) | 0.022 (0.001-0.200) | 1.28 (1.19-1.43) | -296.5 |  |  |
| Papua New Guinea | Senn *et al.*[26] |  | 0.451 (0.224 – 0.792) | 0.053 (0.002-0.346) | 3.11 (2.02-4.69) | -172.9 |  |  |
| Peru | Hayes (2) *et al.*[27] |  | 0.065 (0.040 – 0.116) | 0.149 (0.062-0.344) | 1.58 (1.36-1.99) | -951.6 |  |  |
|  | Reiskind *et al.*[28] |  | 0.022 (0.017 – 0.030) | 0.018 (0.002-0.099) | 1.20 (1.16-1.27) | -692.9 |  |  |
| Singapore | Goh *et al.*[6] |  | 0.089 (0.046 – 0.185) | 0.441 (0.219-0.709) | 1.48 (1.24-2.04) | -176.3 |  |  |
|  | Yew *et al.*[4] |  | 0.040 (0.019 – 0.091) | 0.240 (0.099-0.532) | 1.24 (1.12-1.56) | -68.5 |  |  |
|  | Yap *et al.*[5] |  | 0. 051 (0.031 – 0.090) | 0.167 (0.072-0.373) | 1.32 (1.19-1.56) | -2765.2 |  |  |
| Sri Lanka | Malavige *et al.*[8] |  | 0. 046 (0.036 – 0.060) | 0.025 (0.001-0.113) | 1.26 (1.20-1.35) | -2767.6 |  |  |
|  | Tissera *et al.*[9] |  | 0. 144 (0.125 – 0.166) | 0.006 (0.000-0.0345) | 1.81 (1.67-1.96) | -2440.3 |  |  |
|  | Tam *et al.*[7] |  | 0. 140 (0.127 – 0.160) | 0.004 (0.000-0.025) | 1.78 (1.65-1.93) | -42.3 |  |  |
| Thailand | Perret *et al.*[11] |  | 0.414 (0.116 – 0.942) | 0.139 (0.024-0.556) | 3.96 (1.72-8.33) | -288.1 |  |  |
|  | Tuntaprasart *et al.*[12] |  | 0. 157 (0.118 – 0.204) | 0.033 (0.002-0.164) | 1.96 (1.69-2.28) | -71.9 |  |  |
| USA (Texas) | Brunkard *et al.*[22] |  | 0. 018 (0.011 – 0.030) | 0.071 (0.017-0.258) | 1.09 (1.06-1.15) | -51.1 |  |  |
|  | Ramos *et al.*[23] |  | 0.020 (0.008 – 0.057) | 0.215 (0.055-0.568) | 1.10 (1.01-1.30) | -19.8 |  |  |
| Vietnam | Bartley *et al.*[29] |  | 0.166 (0.117 – 0.232) | 0.120 (0.036-0.293) | 1.85 (1.57-2.21) | -158.8 |  |  |
|  | Thai *et al.*[30] |  | 0.135 (0.105 – 0.176) | 0.035 (0.005-0.164) | 1.76 (1.58-2.01) | -607.5 |  |  |

Assumption 1: tertiary and quaternary infections possible.

**Table S3: Summary parameter estimates where 7 years’ worth of cross-sectional data from Nicaragua were fitted to the time-varying force of infection model (model C).**

| Parameter | Name | Median Estimate (95% CI) | LnL | DIC |
| --- | --- | --- | --- | --- |
| Force of infection | $\lambda$ | 0.323 (0.261 – 0.377) | -7848.2 | 15751.6 |
| Seasonal amplitude | $\delta$ | 0.360 (0.072 – 0.670) |
| Phase Shift | $\theta$ | 0.392 (0.015 – 0.990) |
| Periodicity (yrs) | $T$ | 8.8 (1.3 – 12.5) |
| Scaling of$\lambda$for those under critical age threshold relative to those over that threshold | $S$ | 0.54 (0.39 – 0.84) |
| Critical age (yrs) threshold at which $\lambda$assumed to change | $A_{critical}$ | 3.9 (2.7 – 5.4) |
| Over-dispersion | $\gamma$ | 0.016 (0.009 – 0.028) |

**Table S4: Summary estimates where Model A was fit to PRNT data re-categorised into ‘seronegative’ (PRNT < cut-off for all serotypes) or ‘seropositive’ (PRNT > cut-off for at least one serotype).**

|  |  |  |  | $R_{0i}$ | |  |  |
| --- | --- | --- | --- | --- | --- | --- | --- |
| Country | **Author** | $\lambda$**(95% CI)** | $\gamma$ **(95% CI)** | **Assumption 1 (95% CI)** | **Assumption 2 (95% CI)** | **LnL** | **DIC** |
| Cuba | Guzman *et al.*[33] | 0.040 (0.032 – 0.073) | 0.184 (0.136-0.424 | 1.52 (1.40 – 2.14) | 1.52 (1.40 – 2.14) | -892.7 | 1788.0 |
|  | Guzman *et al.*[34] | 0.014 (0.012 – 0.021) | 0.176 (0.137-0.337) | 1.14 (1.12 – 1.23) | 1.14 (1.12 – 1.23) | 651.8 | 1306.2 |
| Haiti | Halstead *et al.*[35] | 0.398 (0.340 – 0.596) | 0.062 (0.023-0.520) | 3.67 (3.26 – 5.08) | 6.32 (5.49 – 9.16) | -21.5 | 45.2 |
| Indonesia | Graham *et al.*[36] | 0.120 (0.107 – 0.170) | 0.074 (0.047-0.272) | 1.89 (1.79 – 2.32) | 2.81 (2.59 – 3.77) | -1234.0 | 2480.8 |
| Peru | Morrison *et al.*[37] | 0.128 (0.121 – 0.146) | 0.037 (0.028-0.087) | 2.94 (2.82 – 3.28) | 2.94 (2.82 – 3.28) | -1025  4 | 2053.6 |
| Thailand | Sangkawibha *et al.*[38] | 0.170 (0.141 – 0.301) | 0.334 (0.280-0.529) | 2.15 (1.94 – 3.15) | 3.33 (2.86 – 5.45) | -581.2 | 1165.1 |
|  | Rodriguez-Barraquer *et al.*[39] | 0.076 (0.069 – 0.097) | 0.097 (0.073-0.216) | 1.81 (1.73 – 2.09) | 2.77 (2.56 – 3.50) | -1071.2 | 2145.1 |

Assumption 1: tertiary and quaternary infections possible, assumption 2: complete protection after secondary infection.

**Table S5: Summary estimates of the strain-specific forces of infection and reproduction numbers obtained from the fit of PRNT surveys assuming no inter-serotype interaction (model D1).**

|  | **Country** | Cuba | | Haiti | Indonesia | Peru | Thailand |  |  |
| --- | --- | --- | --- | --- | --- | --- | --- | --- | --- |
|  | **Author** | Guzman *et al.*[33] | Guzman *et al.*[34] | Halstead *et al.*[35] | Graham *et al.*[36] | Morrison *et al.*[37] | Sangkawibha *et al.*[38] | Rodriguez-  Barraquer *et al.*[39] | |
| $\lambda_{i}$ | **DENV-1** | 0.027  (0.025 – 0.030) | 0.013  (0.012 – 0.014) | 0.074  (0.030 – 0.129) | 0.046  (0.042 – 0.051) | 0.081  (0.077 – 0.085) | 0.030  (0.023 – 0.038) | 0.024  (0.020 – 0.029) | |
|  | **DENV-2** | 0.013  (0.012 – 0.014) | 0.005  (0.004 – 0.006) | 0.146  (0.094 – 0.204) | 0.058  (0.053 – 0.063) | 0.073  (0.069 – 0.077) | 0.082  (0.072 – 0.093) | 0.041  (0.036 – 0.045) | |
|  | **DENV-3** | NA | NA | 0.103  (0.055 – 0.159) | 0.011  (0.008 – 0.014) | NA | 0.051  (0.042 – 0.060) | 0.024  (0.020 – 0.029) | |
|  | **DENV-4** | NA | NA | 0.167  (0.115 – 0.225) | 0.018  (0.015 – 0.022) | NA | 0.047  (0.038 – 0.056) | 0.029  (0.024 – 0.033) | |
| $\sum_{i} \lambda_{i}$ |  | 0.040  (0.038 – 0.043) | 0.018  (0.017 – 0.020) | 0.494  (0.434 – 0.561) | 0.134  (0.127 – 0.141) | 0.154  (0.148 – 0.160) | 0.209  (0.196 – 0.224) | 0.118  (0.112 – 0.124) | |
| $R_{0i}$ **Assumption 1** | **DENV-1** | 1.93  (1.84 – 2.02) | 1.41  (1.37 – 1.2.02) | 2.97  (1.74 – 4.53) | 2.46  (2.30 – 2.63) | 3.70  (3.54 – 3.86) | 1.79  (1.58 – 2.03) | 2.10  (1.87 – 2.36) | |
|  | **DENV-2** | 1.41  (1.36 – 1.46) | 1.16  (1.13 – 1.16) | 4.99  (3.55 – 6.73) | 2.88  (2.70 – 3.08) | 3.41  (3.27 – 3.55) | 3.36  (3.05 – 3.70) | 3.06  (2.79 – 3.36) | |
|  | **DENV-3** | NA | NA | 3.77  (2.48 – 5.31) | 1.31  (1.23 – 1.40) | NA | 2.40  (2.15 – 2.68) | 2.10  (1.87 – 2.35) | |
|  | **DENV-4** | NA | NA | 5.59  (4.10 – 7.33) | 1.52  (1.42 – 1.63) | NA | 2.28  (2.04 – 2.56) | 2.34  (2.10 – 2.60) | |
| $R_{0i}$ **Assumption 2** | **DENV-1** | 1.93  (1.84 – 2.02) | 1.41  (1.37 – 1.2.02) | 6.91  (6.05 – 7.98) | 3.11  (2.96 – 3.28) | 3.70  (3.54 – 3.86) | 3.56  (3.35 – 3.79) | 4.05  (3.82 – 4.31) | |
|  | **DENV-2** | 1.41  (1.36 – 1.46) | 1.16  (1.13 – 1.16) | 7.80  (6.70 – 9.15) | 3.45  (3.28 – 3.64) | 3.41  (3.27 – 3.55) | 4.42  (4.13 – 4.74) | 4.64  (4.37 – 4.94) | |
|  | **DENV-3** | NA | NA | 7.20  (6.26 – 8.35) | 2.59  (2.48 – 2.71) | NA | 3.80  (3.56 – 4.07) | 4.05  (3.82 – 4.30) | |
|  | **DENV-4** | NA | NA | 8.19  (7.01 – 9.64) | 2.66  (2.54 – 2.79) | NA | 3.75  (3.52 – 4.01) | 4.17  (3.93 – 4.43) | |
|  | **LnL** | -1631.9 | -1091.3 | -137.3 | -2632.8 | -2898.5 | -1320.7 | -1785.2 | |
|  | **DIC** | 3299.9 | 2223.1 | 277.7 | 5299.6 | 5818.7 | 2656.9 | 3602.1 | |

**Table S6: Summary estimates of the force of infection ($\lambda$) and serotype-specific reproduction number ($R_{0i}$) assuming inter-serotype interactions are equal for all serotype combinations (model D2).**

|  | **Country** | Cuba | | Haiti | Indonesia | Peru | Thailand | |
| --- | --- | --- | --- | --- | --- | --- | --- | --- |
|  | **Author** | Guzman *et al.*[33] | Guzman *et al.*[34] | Halstead *et al.*[35] | Graham *et al.*[36] | Morrison *et al.*[37] | Sangkawibha *et al.*[38] | Rodriguez-  Barraquer *et al.*[39] |
| $\lambda_{i}$ | **DENV-1** | 0.027  (0.024 – 0.030) | 0.013  (0.012 – 0.015) | 0.046  (0.010 – 0.179) | 0.042  (0.037 – 0.047) | 0.062  (0.057 – 0.068) | 0.021  (0.015 – 0.029) | 0.012  (0.012 – 0.016) |
|  | **DENV-2** | 0.007  (0.005 – 0.008) | 0.003  (0.002 – 0.004) | 0.120  (0.038 – 0.176) | 0.056  (0.050 – 0.062) | 0.056  (0.050 – 0.063) | 0.081  (0.069 – 0.095) | 0.033  (0.029 – 0.037) |
|  | **DENV-3** | NA | NA | 0.081  (0.023 – 0.373) | 0.008  (0.006 – 0.010) | NA | 0.041  (0.033 – 0.051) | 0.012  (0.009 – 0.016) |
|  | **DENV-4** | NA | NA | 0.219  (0.088 – 0.445) | 0.013  (0.011 – 0.017) | NA | 0.037  (0.029 – 0.047) | 0.018  (0.014 – 0.021) |
| $\sum_{i} \lambda_{i}$ |  | 0.033  (0.031 – 0.036) | 0.016  (0.015 – 0.018) | 0.518  (0.403 – 0.681) | 0.119  (0.112 – 0.127) | 0.118  (0.112 – 0.125) | 0.182  (0.167 – 0.198) | 0.075  (0.070 – 0.080) |
| $\rho$***** |  | 1.350  (0.691 – 2.024) | 0.128  (0.005 – 0.519) | 0.475  (0.107 – 0.994) | 1.100  (0.898 – 1.330) | 0.898  (0.697 – 1.120) | 1.199  (0.935 – 1.493) | 5.561  (4.728 – 6.530) |
| $R_{0i}$ | **DENV-1** | 1.89  (1.75 – 2.07) | 1.50  (1.43 – 1.56) | 10.15  (6.75 – 28.65) | 3.23  (2.99 – 3.51) | 3.67  (3.38 – 3.99) | 3.54  (3.21 – 3.94) | 2.49  (2.29 – 2.71) |
|  | **DENV-2** | 1.32  (1.13 – 1.63) | 1.46  (1.30 – 1.54) | 10.94  (7.33 – 19.03) | 3.50  (3.25 – 3.77) | 3.56  (3.26 – 3.92) | 4.32  (3.92 – 4.79) | 3.25  (3.00 – 3.53) |
|  | **DENV-3** | NA | NA | 10.50  (6.97 – 19.55) | 2.77  (2.56 – 3.02) | NA | 3.76  (3.40 – 4.18) | 2.49  (2.29 – 2.71) |
|  | **DENV-4** | NA | NA | 11.81  (7.83 – 19.57) | 2.84  (2.62 – 3.09) | NA | 3.71  (3.35 – 4.13) | 2.62  (2.40 – 2.84) |
|  | **LnL** | -1631.9 | -1091.3 | -137.3 | -2632.8 | -2885.9 | -1320.7 | -1785.2 |
|  | **DIC** | 3299.9 | 2223.1 | 277.7 | 5299.6 | 5818.7 | 2656.9 | 3602.1 |

*ρ is the interaction parameter describing susceptibility enhancement-inhibition estimated using model D2.

|  |  | Cuba |  | Haiti | Indonesia | Peru | Thailand |  |
| --- | --- | --- | --- | --- | --- | --- | --- | --- |
|  | **Author** | Guzman *et al.*[33] | Guzman *et al.*[34] | Halstead *et al.*[35] | Graham *et al.*[36] | Morrison *et al.*[37] | Sangkawibha *et al.*[38] | Rodriguez-  Barraquer *et al.*[39] |
| $\lambda_{i}$ | **DENV-1** | 0.028  (0.026 – 0.031) | 0.013  (0.012 - 0.015) | 0.116  (0.013 – 0.312) | 0.036  (0.027 – 0.048) | 0.026  (0.022 – 0.030) | 0.014  (0.008 – 0.026) | 0.018  (0.002 – 0.034) |
|  | **DENV-2** | 0.004  (0.003 – 0.006) | 0.003  (0.002 – 0.004) | 0.045  (0.014 – 0.182) | 0.046  (0.038 – 0.060) | 0.091  (0.086 – 0.097) | 0.106  (0.082 – 0.126) | 0.011  (0.007 – 0.024) |
|  | **DENV-3** | NA | NA | 0.115  (0.017 – 0.309) | 0.006  (0.004 – 0.014) | NA | 0.028  (0.019 – 0.046) | 0.021  (0.004 – 0.035) |
|  | **DENV-4** | NA | NA | 0.126  (0.032 – 0.317) | 0.029  (0.016 – 0.041) | NA | 0.030  (0.018 – 0.049) | 0.026  (0.006 – 0.041) |
| $\sum_{i} \lambda_{i}$ |  | 0.033  (0.030 – 0.036) | 0.016  (0.015 – 0.017) | 0.452  (0.358 – 0.579) | 0.119  (0.111 – 0.126) | 0.117  (0.111 – 0.123) | 0.180  (0.165 – 0.196) | 0.075  (0.070 – 0.080) |
| $\rho$ | $\rho_{1\cdot}$ | 4.188  (2.167 – 6.716) | 0.361  (0.018 – 1.414) | 1.785  (0.244 – 12.465) | 0.596  (0.038 – 1.670) | 0.011  (0.000 – 0.053) | 0.397  (0.018 – 1.641) | 9.434  (0.202 – 19.264) |
|  | $\rho_{2\cdot}$ | 0.126  (0.005 – 0.736) | 0.143  (0.005 – 0.719) | 0.237  (0.013 – 1.065) | 0.333  (0.013 – 1.345) | 4.904  (3.736 – 6.291) | 2.781  (1.492 – 4.612) | 0.653  (0.031 – 3.294) |
|  | $\rho_{3\cdot}$ | NA | NA | 1.095  (0.150 – 5.693) | 0.464  (0.018 – 2.789) | NA | 0.473  (0.029 – 1.526) | 11.475  (1.227 – 19.503) |
|  | $\rho_{4\cdot}$ | NA | NA | 0.510  (0.052 – 2.011) | 4.277  (1.804 – 7.217) | NA | 0.759  (0.080 – 1.978) | 9.644  (1.374 – 19.101) |
| $R_{0i}$ | **DENV-1** | 1.41  (1.17 – 1.72) | 1.47  (1.35 – 1.47) | 5.08  (1.13 – 12.07) | 3.71  (2.30 – 5.12) | 5.02  (4.78 – 5.26) | 5.03  (2.77 – 6.63) | 1.46  (1.06 – 5.20) |
|  | **DENV-2** | 2.02  (1.61 – 2.19) | 1.45  (1.23 – 1.54) | 11.94  (7.10 – 17.58) | 4.43  (2.88 – 5.25) | 1.43  (1.20 – 1.72) | 2.48  (1.49 – 3.79) | 5.03  (3.74 – 5.62) |
|  | **DENV-3** | NA | NA | 7.17  (2.47 – 13.78) | 3.59  (1.34 – 5.17) | NA | 4.99  (3.13 – 6.59) | 1.24  (1.04 – 4.02) |
|  | **DENV-4** | NA | NA | 10.52  (5.90 – 16.49) | 1.55  (1.00 – 2.08) | NA | 4.25  (2.57 – 6.29) | 1.45  (1.08 – 4.05) |
|  | LNL | -1625.4 | 1091.8 | -136.7 | -2629.1 | -2804.8 | -1316.3 | -1779 |
|  | DIC | 3278.9 | 2221.2 | 276.5 | 5293.9 | 5632 | 2647.6 | 3582.3 |

**Table S7: Summary estimates assuming inter-serotype interactions are dependent only on the primary infecting serotype (model D3).**

**Table S8: Summary estimates assuming inter-serotype interactions are dependent only on the secondary infecting serotype (model D4).**

|  | **Country** | **Cuba** | | **Haiti** | **Indonesia** | **Peru** | **Thailand** | |
| --- | --- | --- | --- | --- | --- | --- | --- | --- |
|  | **Author** | Guzman *et al.*[33] | Guzman *et al.*[34] | Halstead *et al.*[35] | Graham *et al.*[36] | Morrison *et al.*[37] | Sangkawibha *et al.*[38] | Rodriguez-Barraquer *et al.*[39] |
| $\lambda_{i}$ | **DENV-1** | 0.028  (0.026 – 0.031) | 0.013  (0.012 – 0.015) | 0.035  (0.008 – 0.174) | 0.044  (0.033 – 0.053) | 0.026  (0.022 – 0.030) | 0.017  (0.011 – 0.026) | 0.013  (0.009 – 0.018) |
|  | **DENV-2** | 0.004  (0.003 – 0.006) | 0.003  (0.002 – 0.004) | 0.076  (0.023 – 0.267) | 0.055  (0.044 – 0.067) | 0.092  (0.086 – 0.097) | 0.096  (0.078 – 0.113) | 0.029  (0.024 – 0.039) |
|  | **DENV-3** | NA | NA | 0.077  (0.016 – 0.313) | 0.007  (0.005 – 0.010) | NA | 0.035  (0.023 – 0.048) | 0.013  (0.009 – 0.019) |
|  | **DENV-4** | NA | NA | 0.208  (0.056 – 0.401) | 0.013  (0.010 – 0.017) | NA | 0.033  (0.022 – 0.046) | 0.019  (0.014 – 0.027) |
| $\sum_{i} \lambda_{i}$ |  | 0.033  (0.030 – 0.036) | 0.016  (0.015 – 0.017) | 0.456  (0.363 – 0.575) | 0.119  (0.111 – 0.126) | 0.117  (0.111 – 0.124) | 0.180  (0.165 – 0.196) | 0.075  (0.071 – 0.080) |
| $\rho$ | $\rho_{\cdot1}$ | 0.125  (0.005 – 0.699) | 0.142  (0.005 – 0.705) | 1.061  (0.026 – 8.135) | 0.558  (0.021 – 2.443) | 4.909  (3.735 – 6.313) | 3.030  (0.138 – 8.914) | 5.287  (0.343 – 9.690) |
|  | $\rho_{\cdot2}$ | 4.158  (2.173 – 6.727) | 0.359  (0.016 – 1.444) | 1.322  (0.056 – 8.146) | 1.061  (0.066 – 2.882) | 0.011  (0.000 – 0.052) | 0.187  (0.008 – 0.965) | 7.975  (3.015 – 9.929) |
|  | $\rho_{\cdot3}$ | NA | NA | 0.479  (0.013 – 5.901) | 2.774  (0.106 – 9.050) | NA | 1.793  (0.100 – 5.862) | 4.838  (0.319 – 9.715) |
|  | $\rho_{\cdot4}$ | NA | NA | 0.372  (0.014 – 3.571) | 0.912  (0.038 – 4.090) | NA | 1.260  (0.054 – 5.463) | 4.408  (0.321 – 9.570) |
| $R_{0i}$ | **DENV-1** | 1.41  (1.17 – 1.71) | 1.47  (1.35 – 1.54) | 7.44  (4.46 – 12.44) | 2.82  (2.00 – 4.08) | 5.03  (4.80 – 5.27) | 3.65  (2.38 – 5.22) | 2.27  (1.95 – 2.78) |
|  | **DENV-2** | 2.02  (1.63 – 2.19) | 1.45  (1.24 – 1.53) | 9.04  (5.42 – 14.24) | 3.39  (2.39 – 4.63) | 1.43  (1.20 – 1.73) | 3.10  (2.32 – 3.95) | 3.46  (2.84 – 3.93) |
|  | **DENV-3** | NA | NA | 8.01  (3.86 – 13.77) | 1.99  (1.07 – 3.47) | NA | 3.27  (2.08 – 5.12) | 2.56  (1.98 – 3.41) |
|  | **DENV-4** | NA | NA | 7.86  (4.56 – 13.27) | 2.69  (1.98 – 3.50) | NA | 3.42  (2.43 – 5.17) | 2.33  (1.97 – 2.98) |
|  | LNL | -1625.4 | -1091.8 | -137.1 | -2632.7 | -2804.8 | -1317.6 | -1784.6 |
|  | DIC | 3283.3 | 2219.5 | 274.3 | 5298 | 5632.9 | 2649.4 | 3596.9 |

**Table S9: DIC comparison of different model variants (A, D1 – D4) for serotype-specific PRNT datasets.**

| **Author/Country/Ref** | **Model Variant DIC** |  |  |  |  |
| --- | --- | --- | --- | --- | --- |
|  | **A** | **D1** | **D2** | **D3** | **D4** |
| **Guzman/Cuba/**[33] | 1788.0 | 3299.9 | 3299.9 | 3278.9 | 3283.3 |
| **Guzman/Cuba/**[34] | 1306.2 | 2223.1 | 2223.1 | 2221.2 | 2219.5 |
| **Halstead/Haiti/**[35] | 45.2 | 277.7 | 277.7 | 276.5 | 274.3 |
| **Graham/Indonesia/**[36] | 2480.8 | 5299.6 | 5299.6 | 5293.9 | 5298 |
| **Morrison/Peru/**[37] | 2053.6 | 5818.7 | 5818.7 | 5632 | 5632.9 |
| **Sangkawibha/Thailand/**[38] | 1165.1 | 2656.9 | 2656.9 | 2647.6 | 2649.4 |
| **Rodriguez-Barraquer/Thailand/**[39] | 2145.1 | 3602.1 | 3602.1 | 3582.3 | 3596.9 |

**References:**

1. Ferguson NM, Donnelly CA, Anderson RM (1999) Transmission dynamics and epidemiology of dengue: insights from age-stratified sero-prevalence surveys. Philos Trans R Soc London Ser B-Biological Sci 354: 757–768. doi:10.1098/rstb.1999.0428.

2. Gubler DJ, Suharyono W, Tan R, Abidin M, Sie A (1981) Viraemia in patients with naturally acquired dengue infection. Bull World Health Organ 59: 623–630.

3. Vaughn D, Green S (2000) Dengue viremia titer, antibody response pattern, and virus serotype correlate with disease severity. J Infect Dis: 2–9.

4. Yew YW, Ye T, Ang LW, Ng LC, Yap G, et al. (2009) Seroepidemiology of dengue virus infection among adults in Singapore. Ann Acad Med Singapore 38: 667–675.

5. Yap G, Li C, Mutalib A, Lai Y-L, Ng L-C (2013) High rates of inapparent dengue in older adults in Singapore. Am J Trop Med Hyg 88: 1065–1069. doi:10.4269/ajtmh.12-0150.

6. Goh KT, Yamazaki S (1987) Serological survey on dengue virus infection in Singapore. Trans R Soc Trop Med Hyg 81: 687–689. doi:10.1016/0035-9203(87)90456-1.

7. Tam CC, Tissera H, de Silva AM, De Silva AD, Margolis HS, et al. (2013) Estimates of dengue force of infection in children in Colombo, Sri Lanka. PLoS Negl Trop Dis 7: e2259. doi:10.1371/journal.pntd.0002259.

8. Malavige GN, Fernando S, Aaskov J, Sivayogan S, Dissanayaka T, et al. (2006) Seroprevalence of anti-dengue virus antibodies in children in Colombo District, Sri Lanka. Dengue Bull 30: 12–68.

9. Tissera HA, De Silva AD, Abeysinghe MRN, de Silva AM, Palihawadana P, et al. (2010) Dengue Surveillance in Colombo, Sri Lanka: Baseline seroprevalence among children. Procedia Vaccinol 2: 107–110.

10. Padbidri VS, Wairagkar NS, Joshi GD, Umarani UB, Risbud AR, et al. (2002) A serological survey of arboviral diseases among the human population of the Andaman and Nicobar Islands, India. Southeast Asian J Trop Med Public Health 33: 794–800.

11. Perret C, Chanthavanich P, Pengsaa K, Limkittikul K, Hutajaroen P, et al. (2005) Dengue infection during pregnancy and transplacental antibody transfer in Thai mothers. J Infect 51: 287–293. doi:10.1016/j.jinf.2004.10.003.

12. Tuntaprasart W, Barbazan P, Nitatpattana N, Rongsriyam Y, Yoksan S, et al. (2003) Seroepidemiological survey among schoolchildren during the 2000-2001 dengue outbreak of Ratchaburi Province, Thailand. Southeast Asian J Trop Med Public Health 34: 564–568.

13. Vallee J, Dubot-Peres A, Ounaphom P, Sayavong C, Bryant JE, et al. (2009) Spatial distribution and risk factors of dengue and Japanese encephalitis virus infection in urban settings: The case of Vientiane, Lao PDR. Trop Med Int Heal 14: 1134–1142. doi:http://dx.doi.org/10.1111/j.1365-3156.2009.02319.x.

14. Hiscox A, Winter CH, Vongphrachanh P, Sisouk T, Somoulay V, et al. (2010) Short Report Serological Investigations of Flavivirus Prevalence in Khammouane Province, Lao People’s Democratic Republic, 2007-2008. Am J Trop Med Hyg 83: 1166–1169. doi:10.4269/ajtmh.2010.09.0480.

15. Braga C, Luna CF, Martelli CM, de Souza W V, Cordeiro MT, et al. (2010) Seroprevalence and risk factors for dengue infection in socio-economically distinct areas of Recife, Brazil. Acta Trop 113: 234–240. doi:10.1016/j.actatropica.2009.10.021.

16. Iturrino-Monge R, Avila-Agüero ML, Avila-Agüero CR, Moya-Moya T, Cañas-Coto A, et al. (2006) Seroprevalence of dengue virus antibodies in asymptomatic Costa Rican children, 2002-2003: a pilot study. Rev Panam Salud Pública 20: 39–43. doi:10.1590/S1020-49892006000700005.

17. Yamashiro T, Disla M, Petit A, Taveras D, Castro-Bello M, et al. (2004) Seroprevalence of IgG specific for dengue virus among adults and children in Santo Domingo Dominican Republic. Am J Trop Med Hyg 71: 138–143.

18. Hayes JM, García-Rivera E, Flores-Reyna R, Suárez-Rangel G, Rodríguez-Mata T, et al. (2003) Risk factors for infection during a severe dengue outbreak in El Salvador in 2000. Am J Trop Med Hyg 69: 629–633.

19. Deparis X, Roche C, Murgue B, Chungue E (1998) Possible dengue sequential infection: dengue spread in a neighbourhood during the 1996/97 dengue-2 epidemic in French Polynesia. Trop Med Int Heal 3: 866–871.

20. Hiscox A, Winter CH, Vongphrachanh P, Sisouk T, Somoulay V, et al. (2010) Serological investigations of flavivirus prevalence in Khammouane Province, Lao People’s Democratic Republic, 2007-2008. Am J Trop Med Hyg 83: 1166–1169. doi:10.4269/ajtmh.2010.09-0480.

21. Sissoko D, Ezzedine K, Giry C, Moendandze A, Lernout T, et al. (2010) Seroepidemiology of Dengue virus in Mayotte, Indian Ocean, 2006. PLoS One 5: e14141. doi:10.1371/journal.pone.0014141.

22. Brunkard JM, Robles Lopez JL, Ramirez J, Cifuentes E, Rothenberg SJ, et al. (2007) Dengue fever seroprevalence and risk factors, Texas-Mexico border, 2004. Emerg Infect Dis 13: 1477–1483. doi:10.3201/eid1310.061586.

23. Ramos MM, Mohammed H, Zielinski-Gutierrez E, Hayden MH, Lopez JL, et al. (2008) Epidemic dengue and dengue hemorrhagic fever at the Texas-Mexico border: results of a household-based seroepidemiologic survey, December 2005. Am J Trop Med Hyg 78: 364–369.

24. Ali A, Rehman HU, Nisar M, Rafique S, Ali S, et al. (2013) Seroepidemiology of dengue fever in Khyber Pakhtunkhawa, Pakistan. Int J Infect Dis 17: E518–E523. doi:10.1016/j.ijid.2013.01.007.

25. Mahmood S, Nabeel H, Hafeez S, Zahra U, Nazeer H (2013) Seroprevalence of Dengue IgG Antibodies among Healthy Adult Population in Lahore, Pakistan. ISRN Trop Med 2013: 1–6. doi:10.1155/2013/521396.

26. Senn N, Luang-Suarkia D, Manong D, Siba PM, McBride WJ (2011) Contribution of dengue fever to the burden of acute febrile illnesses in Papua New Guinea: an age-specific prospective study. Am J Trop Med Hyg 85: 132–137. doi:10.4269/ajtmh.2011.10-0482.

27. Hayes CG, Phillips IA, Callahan JD, Griebenow WF, Hyams KC, et al. (1996) The epidemiology of dengue virus infection among urban, jungle, and rural populations in the Amazon region of Peru. Am J Trop Med Hyg 55: 459–463.

28. Reiskind MH, Baisley KJ, Calampa C, Sharp TW, Watts DM, et al. (2001) Epidemiological and ecological characteristics of past dengue virus infection in Santa Clara, Peru. Trop Med Int Heal 6: 212–218. doi:10.1046/j.1365-3156.2001.00703.x.

29. Bartley LM, Carabin H, Vinh Chau N, Ho V, Luxemburger C, et al. (2002) Assessment of the factors associated with flavivirus seroprevalence in a population in Southern Vietnam. Epidemiol Infect 128: 213–220. doi:10.1017/s0950268801006495.

30. Thai KTD, Binh TQ, Giao PT, Phuong HL, Hung le Q, et al. (2005) Seroprevalence of dengue antibodies, annual incidence and risk factors among children in southern Vietnam. Trop Med Int Heal 10: 379–386. doi:10.1111/j.1365-3156.2005.01388.x.

31. Balmaseda A, Hammond SN, Tellez Y, Imhoff L, Rodriguez Y, et al. (2006) High seroprevalence of antibodies against dengue virus in a prospective study of schoolchildren in Managua, Nicaragua. Trop Med Int Heal 11: 935–942. doi:10.1111/j.1365-3156.2006.01641.x.

32. Balmaseda A, Standish K, Mercado JC, Matute JC, Tellez Y, et al. (2010) Trends in patterns of dengue transmission over 4 years in a pediatric cohort study in Nicaragua. J Infect Dis 201: 5–14. doi:10.1086/648592.

33. Guzman MG, Kouri GP, Bravo J, Soler M, Vazquez S, et al. (1990) DENGUE HEMORRHAGIC-FEVER IN CUBA, 1981 - A RETROSPECTIVE SEROEPIDEMIOLOGIC STUDY. Am J Trop Med Hyg 42: 179–184.

34. Guzman MG, Kouri G, Valdes L, Bravo J, Alvarez M, et al. (2000) Epidemiologic studies on dengue in Santiago de Cuba, 1997. Am J Epidemiol 152: 793–799. doi:10.1093/aje/152.9.793.

35. Halstead SB, Streit TG, Lafontant JG, Putvatana R, Russell K, et al. (2001) Haiti: Absence of dengue hemorrhagic fever despite hyperendemic dengue virus transmission. Am J Trop Med Hyg 65: 180–183.

36. Graham RR, Juffrie M, Tan R, Hayes CG, Laksono I, et al. (1999) A prospective seroepidemiologic study on dengue in children four to nine years of age in Yogyakarta, Indonesia I. studies in 1995-1996. Am J Trop Med Hyg 61: 412–419.

37. Morrison AC, Minnick SL, Rocha C, Forshey BM, Stoddard ST, et al. (2010) Epidemiology of dengue virus in Iquitos, Peru 1999 to 2005: interepidemic and epidemic patterns of transmission. PLoS Neglected Trop Dis [electronic Resour 4: e670. doi:10.1371/journal.pntd.0000670.

38. Sangkawibha N, Rojanasuphot S, Ahandrik S, Viriyapongse S, Jatanasen S, et al. (1984) RISK-FACTORS IN DENGUE SHOCK SYNDROME - A PROSPECTIVE EPIDEMIOLOGIC-STUDY IN RAYONG, THAILAND .1. THE 1980 OUTBREAK. Am J Epidemiol 120: 653–669.

39. Rodriguez-Barraquer I, Buathong R, Iamsirithaworn S, Nisalak A, Lessler J, et al. (2013) Revisiting Rayong: Shifting Seroprofiles of Dengue in Thailand and Their Implications for Transmission and Control. Am J Epidemiol 179: 353–360. doi:10.1093/aje/kwt256.
